# GEEES: inferring cell-specific gene–enhancer interactions from multi-modal single-cell data

**DOI:** 10.1093/bioinformatics/btae638

**Published:** 2024-10-28

**Authors:** Shuyang Chen, Sündüz Keleş

**Affiliations:** Department of Statistics, University of Wisconsin-Madison, Madison, WI 53706, United States; Department of Statistics, University of Wisconsin-Madison, Madison, WI 53706, United States; Department of Biostatistics and Medical Informatics, University of Wisconsin-Madison, Madison, WI 53706, United States

## Abstract

**Motivation:**

Gene–enhancer interactions are central to transcriptional regulation. Current multi-modal single-cell datasets that profile transcriptome and chromatin accessibility simultaneously in a single cell are yielding opportunities to infer gene–enhancer associations in a cell type specific manner. Computational efforts for such multi-modal single-cell datasets thus far focused on methods for identification and refinement of cell types and trajectory construction. While initial attempts for inferring gene–enhancer interactions have emerged, these have not been evaluated against benchmark datasets that materialized from bulk genomic experiments. Furthermore, existing approaches are limited to inferring gene–enhancer associations at the level of grouped cells as opposed to individual cells, thereby ignoring regulatory heterogeneity among the cells.

**Results:**

We present a new approach, GEEES for “**G**ene **E**nhanc**E**r Int**E**ractions from Multi-modal **S**ingle Cell Data,” for inferring gene–enhancer associations at the single-cell level using multi-modal single-cell transcriptome and chromatin accessibility data. We evaluated GEEES alongside several multivariate regression-based alternatives we devised and state-of-the-art methods using a large number of benchmark datasets, providing a comprehensive assessment of current approaches. This analysis revealed significant discrepancies between gold-standard interactions and gene–enhancer associations derived from multi-modal single-cell data. Notably, incorporating gene–enhancer distance into the analysis markedly improved performance across all methods, positioning GEEES as a leading approach in this domain. While the overall improvement in performance metrics by GEEES is modest, it provides enhanced cell representation learning which can be leveraged for more effective downstream analysis. Furthermore, our review of existing experimentally driven benchmark datasets uncovers their limited concordance, underscoring the necessity for new high-throughput experiments to validate gene–enhancer interactions inferred from single-cell data.

**Availability and implementation:**

https://github.com/keleslab/GEEES.

## 1 Introduction

Gene regulation with DNA *cis*-regulatory elements, such as enhancers and promoters, is the basis of many biological processes including development, differentiation, immunity, homeostasis, and disease (Cusanovich *et al.* 2018, Rickels and Shilatifard 2018, Domcke *et al.* 2020). Single modality single-cell technologies thus far enabled investigation of gene regulatory mechanisms from the point of transcriptomics (Rickels and Shilatifard 2018), epigenomics (Kundaje *et al.* 2015), or 3D structures (Ramani *et al.* 2017, Tan 2021, Li *et al.* 2023), while creating opportunities for inferring gene–enhancer interactions (Pliner *et al.* 2018). More recently, emerging multi-modal single-cell technologies such as sci-CAR (Cao *et al.* 2018), SNARE-seq (Chen *et al.* 2019), SHARE-seq (Ma *et al.* 2020), and ISSAAC-seq (Xu *et al.* 2022) that profile both transcriptome and chromatin accessibility within the same cell accelerated these efforts. While such multi-modal experiments initially yielded somewhat lower quality measurements and/or lower throughput compared to their single modality counterparts (Bravo González-Blas *et al.* 2020), the Single-Cell Multiome ATAC + Gene Expression kit (De Rop *et al.* 2024) from 10X Genomics achieved comparable throughput as their single modality counterparts (i.e. single modality scRNA-seq and scATAC-seq), instigating rapid improvement in quality and growing potential for gene–enhancer interaction inference. Initial computational approaches for multi-modal scRNA-seq and scATAC-seq datasets and integrative approaches for their single modality counterparts focused on identification and refinement of cell and sub-cell types and trajectory construction [as reviewed in Baysoy *et al.* (2023)], while advancement of approaches for detecting gene–enhancer interactions appeared secondary. Current methods for identifying gene–enhancer interactions from multi-modal single-cell data can be divided into two classes depending on whether or not they originate from integrative pipelines of multi-modal single-cell data [e.g. methods benchmarked in scIB (Luecken *et al.* 2022) for scRNA-seq and scATAC-seq]. Since integrated multi-modal single-cell data generate cell groups in the two domains that can be considered as coupled, methods such as FigR (Kartha *et al.* 2022) and snapATAC (Fang *et al.* 2021) developed in this realm, which are similar to the marginal method benchmarked in this paper (Supplementary Table S1), are applicable to multi-modal single-cell data. Additionally, several methods are developed specifically for multi-modal single-cell data with measurements of gene expression and chromatin accessibility from the same cell (summarized in Supplementary Table S1). In their pioneering SHARE-seq paper, Ma *et al.* (2020) adapted a method based on *marginal correlation* analysis of expression of individual genes and chromatin accessibility of candidate enhancers by utilizing cells as experimental units. This approach used enhancer-specific resampling-based background null distributions to take into account the local characteristics of individual enhancers, and inferred gene–enhancer pairs with significant marginal correlation as interacting. TRIPOD (Jiang *et al.* 2022) emerged as a computational framework to identify enhancer-transcription factor (TF)-gene trios and link enhancers to genes with mediating transcription factors. scREG (Duren *et al.* 2022) utilized dimension reduction and the concept of *cis*-regulatory potential and inferred latent embeddings to capture gene expression, chromatin accessibility, and their regulatory relationship. More recently, *marginal correlation* approach was applied with metacells to alleviate sparsity issues common to single-cell data (Xie *et al.* 2023). Furthermore, SCARlink (Mitra *et al.* 2024), which employs regularized Poisson regression, presented the earliest multivariate regression model, while this work was under review. There are two apparent shortcomings of the existing studies. First, all methods focus on identifying gene–enhancer interactions, at best, by treating a group of cells (e.g. cells that conform to a cell type) as homogeneous, and ignore the potential heterogeneity of regulatory patterns within cell sub-populations. This hinders capturing of regulatory dynamics within a cell type, where genes are dynamically regulated by different enhancers in development (Yokoshi and Fukaya 2019). Second, and more importantly, these methods often emerged as part of larger studies and were evaluated with different metrics, experimental setups, and benchmark datasets, making it challenging to compare and select among them. While there is a large repertoire of gene–enhancer interactions established using bulk experimental techniques (Bhattacharyya *et al.* 2019, Gao and Qian 2020, Moore *et al.* 2020), there is no systematic comparison of gene–enhancer interactions inferred from single-cell datasets to these benchmarks.

To address the potential heterogeneity of regulatory patterns at the cell level, we propose a new computational method GEEES (cell specific **G**ene **E**nhanc**E**r int**E**ractions from multi-modal **S**ingle cell data) to infer cell specific *cis*-regulatory interactions from multi-modal single-cell data. GEEES estimates gene–enhancer associations at the single-cell level by considering a cell neighborhood defined by both the expression of the gene and the accessibility of the enhancer in the gene–enhancer pair. We benchmarked GEEES against the state-of-the-art methods and a number of multivariate regression approaches we devised on a diverse set of multi-modal single-cell datasets using a wide variety of gold standard gene–enhancer interaction datasets. Surprisingly, these comparisons revealed poor performance of all the methods in terms of standard evaluation metrics [i.e. area under the receiving operating characteristics curve (AUROC), area under the precision–recall curve (AUPR)] with these gold standard datasets, revealing the limits of validating such interactions inferred from single-cell data with the gene–enhancer interactions identified through bulk high throughput experiments. In exploring this observation further, we discovered that while aggregating cells into metacells to alleviate sparsity issues did not improve the performance of the methods, an adjustment based on the distances between the gene–enhancer pairs yielded marked improvements. A detailed investigation of the gold standard datasets revealed sub-optimal concordance between them and highlighted that gene–enhancer pairs that appeared in the gold standard datasets tended to have shorter genomic distances. This underscores the critical necessity for “ground truth” experimental data to verify gene–enhancer connections inferred from single-cell technologies and benchmark the emerging methods. While GEEES provided a slight but consistent improvement over the existent methods across the benchmark experiments, the data reduction it provided in the form of a cells by gene–enhancer interactions matrix uncovered heterogeneity of regulatory patterns of CD14+ monocytes.

## 2 Materials and methods

### 2.1 GEEES: gene–enhancer interactions from multi-modal single-cell data

**Figure 1.**
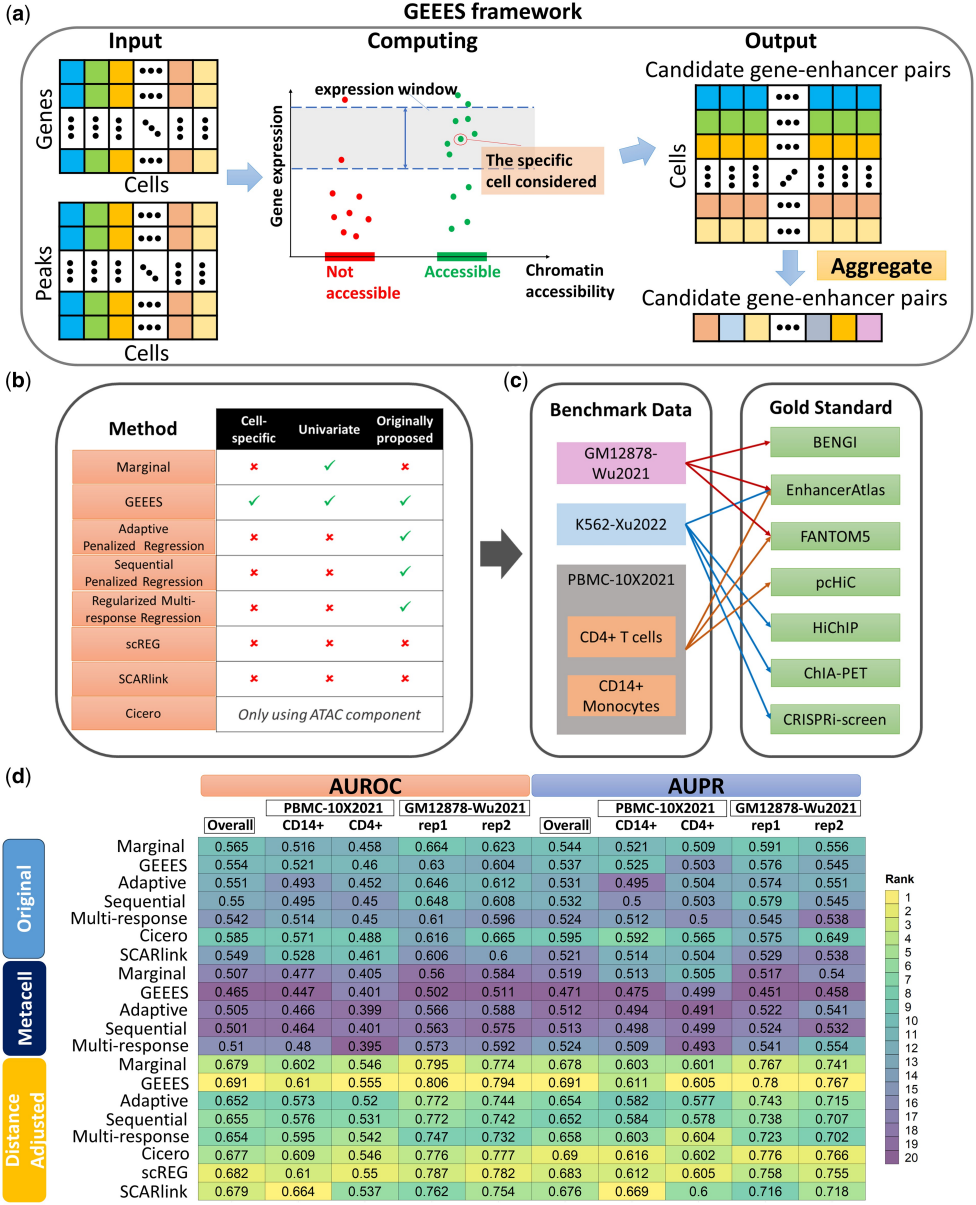
Overview of GEEES and the benchmarking pipeline, and the overall summary of the results. (a) GEEES takes as input a gene (G) by cell (C) gene expression matrix and a peak (*E*) by cell (*C*) chromatin accessibility matrix from a multi-modal single-cell dataset. It outputs a cell (C) by candidate gene–enhancer pairs ($\sum_{g}^{G}E_{g}$) matrix with scores quantifying the strength of the association for each single cell. This matrix is further aggregated across cells to reveal the gene–enhancer interactions in the sample. (b) Overview of the benchmarking pipeline. The methods listed were also evaluated using both metacells and a distance adjustment together with scREG. (c) Test datasets used for benchmarking and their corresponding gold standard datasets for evaluation. FANTOM5 for lymphocyte of B lineage is used for evaluation in GM12878-Wu2021. (d) Overall summary of the benchmarking experiments, both for individual datasets and in aggregate (Overall). The rankings of methods in each dataset are displayed along the columns with the numerical performance metrics. The numerical performance metrics are calculated based on aggregated gold standard where a gene–enhancer pair is true positive if it is validated by any gold standard for that dataset. Here, the benchmark results of K562-Xu2022 are excluded due to the lack of fragments file which is necessary for SCARlink. The overall summary including K562-Xu2022, while excluding SCARlink is provided in Supplementary Fig. S1.

The GEEES method (Fig. 1a) is inspired by the recently developed CSN method (Dai *et al.* 2019) that constructs cell-specific networks from scRNA-seq data to address potential cell-level heterogeneity and nonlinearity in gene–gene associations. In order to quantify the association between a gene g and a candidate enhancer e based on multi-modal single-cell data in a population of cells, GEEES involves three main steps.

First, for each single cell c, a cell neighborhood is constructed with a predefined size k (default k=30). This construction ensures that cells in the neighborhood of cell c for gene g have expression values similar to that of cell c’s expression for gene g.

Next, a Binomial test is employed to evaluate whether the accessibility of a given candidate enhancer e in the group of $n_{cg}$ neighbor cells is on par with the average accessibility (${\hat{\pi }}_{e}$) of that enhancer across all the cells in the sample. Considering $N_{\mathrm{cge}}$, the number of neighborhood cells accessible at enhancer e, this test can be formally carried out for cell c by a Binomial test where $N_{\mathrm{cge}}∼\text{Binomial}(n_{cg},{\hat{\pi }}_{e})$ under the null hypothesis. While the size of the neighborhood $n_{cg}$ can be set adaptively, we set $n_{cg}=k$ in this article due to computational reasons (Wang *et al.* 2021). This testing results in cell and gene–enhancer pair specific *P*-values $p_{\mathrm{cge}}$.

Finally, a summary score $s_{ge}$ for the gene–enhancer pair is obtained by aggregating the cell-specific *P*-values using median aggregation as $s_{ge}=-{\text{median}}_{c\in \{1,..,C\}}{\;\text{log}\;}_{10}(p_{\mathrm{cge}})$. This resulting statistic is utilized for gene–enhancer interaction thresholding and generating the AUROC and AUPR curves.

### 2.2 Sparse multivariate regression models

The gene–enhancer interaction problem can naturally be cast as a variable selection problem within a multivariate regression framework. Here, we considered three sparse linear regression models with additional features that utilized data characteristics.

#### 2.2.1 Adaptive penalized regression

Our first regression formulation is based on adaptive lasso regression (Zou 2006) which induces sparsity on the regression coefficients while allowing modulation of the sparsity levels on the individual regression coefficients. We consider the gene-specific model $Y_{cg}=\beta_{g0}+\sum_{e=1}^{E_{g}}\beta_{ge}A_{ce}+\epsilon_{\mathrm{cge}}$, where $Y_{cg}$ denotes expression of gene g in cell c, $A_{ce}$ accessibility of enhancer e in cell c, and $E_{g}$ denotes the set of candidate enhancers for gene g. Adaptive lasso regression solves the following optimization problem with the penalty function of sum of the weighted $\ell_{1}$-norm of the coefficients $\beta_{ge}$:

(1) $$\begin{array}{c} \underset{{\left\{\beta_{ge}\right\}}_{e=0}^{E_{g}}}{\min}\frac{1}{C}\sum_{c=1}^{C}{\left[Y_{cg}-\left(\beta_{g0}+\sum_{e=1}^{E_{g}}\beta_{ge}A_{ce}\right)\right]}^{2}\\ +\lambda \sum_{e=1}^{E_{g}}\phi_{ge}||\beta_{ge}||. \end{array}$$

Here, λ is a regularization parameter and $\phi_{ge}$ is a gene–enhancer specific adaptive weight. We set the weight $\phi_{ge}$ to be inversely proportional to the co-accessibility score between gene g and enhancer e. The co-accessibility score is estimated by the cicero package (Pliner *et al.* 2018) which quantifies the association between the promoter of the gene and the enhancer by utilizing only the scATAC-seq component of the data. This aims to penalize enhancers that are co-accessible with the promoter of gene g less. In addition, cicero is also benchmarked in this study.

#### 2.2.2 Sequential penalized regression

As an alternative to the *adaptive penalized regression*, we considered reducing the dimensionality, i.e. reduction on $E_{g}$, g=1,…,G, with a sequential approach that employed two Lasso regressions. The first Lasso formulation regressed accessibility of promoter of gene g on the accessibility of the candidate enhancers. Subsequently, the second formulation applied Lasso regression on the subset of enhancers with positive coefficients from the first step. This first Lasso regression formulation of this approach aims to replace the adaptive penalty in Equation (1) with an explicit selection step.

#### 2.2.3 Regularized multi-response regression

Combining the two Lasso formulations in the *sequential penalized regression*, we also considered a *multi-response* approach (Peng *et al.* 2010) where we simultaneously regressed the promoter accessibility and the expression of gene g on its candidate enhancers. Specifically, we considered the following penalized formulation that both induces overall sparsity and also groups the coefficients of an enhancer that contribute to association with both the gene promoter accessibility and the gene expression:

$$\begin{array}{c} \underset{{\left\{\alpha_{ge},\beta_{ge}\right\}}_{e=0}^{E_{g}}}{\min}\frac{1}{C}\sum_{i=1}^{C}{\left[Y_{cg}-\left(\beta_{g0}+\sum_{e=1}^{E_{g}}\beta_{ge}A_{ce}\right)\right]}^{2}\\ \;+\frac{1}{C}\sum_{i=1}^{C}{\left[A_{cg}^{*}-\left(\alpha_{g0}+\sum_{e=1}^{E_{g}}\alpha_{ge}A_{ce}\right)\right]}^{2}\\ \;+\lambda_{1}\left(\sum_{e=1}^{E_{g}}||\beta_{ge}||+||\alpha_{ge}||\right)\\ \;+\lambda_{2}\sum_{e=1}^{E_{g}}\sqrt{||\beta_{ge}|{|}^{2}+||\alpha_{ge}|{|}^{2}}, \end{array}$$

where $A_{cg}^{*}$ denotes the promoter accessibility of gene g in cell c, and $\lambda_{1}$ and $\lambda_{2}$ are regularization parameters. Here, the ℓ-1 penalty induces overall sparsity to promote enhancer selection for gene g and the ℓ-2 penalty promotes grouping, and encourages $\beta_{ge}$ and $\alpha_{ge}$, which quantify contribution of accessibility of enhancer e on promoter accessibility and expression of gene g, respectively, to be estimated concordantly.

To enhance the robustness of the regression approaches, all regression methods were implemented with the *stability selection* framework (Meinshausen and Bühlmann 2010), which provides finite sample family wise multiple testing error control and less dependency on the regularization parameter choices. With *stability selection*, the regression model is fit on 100 random subsamples of size ⌊C/2⌋ from the original data with a wide range for the regularization parameters (λ, or $\lambda_{1}$ and $\lambda_{2}$ in Supplementary Section S2) and the empirical selection probability of each enhancer is quantified across these fits. These selection rates of enhancers are then utilized for downstream analysis including gene–enhancer interaction thresholding and generation of the AUROC and AUPR curves.

### 2.3 Existing approaches considered in the benchmarking experiments

#### 2.3.1 Marginal correlation approach

We used the R package Signac (Stuart *et al.* 2021) to implement the *marginal correlation* approach of Ma *et al.* (2020) which quantifies a gene–enhancer association by the Spearman correlation between the expression of the gene and the accessibility of the enhancer across cells. A key feature of this approach is that it evaluates the significance of the resulting associations based on a gene–enhancer specific null distribution that takes into account inherent biases of the sequencing data. The negative of the resulting p-value is utilized for gene–enhancer interaction thresholding and in generation of the AUROC and AUPR curves. Since the gene–enhancer identification pipelines of FigR (Kartha *et al.* 2022) and snapATAC (Fang *et al.* 2021) both employ the marginal association approach, the benchmarking results for *marginal correlation* also highlight the performances of these two methods.

#### 2.3.2 Metacell-based analysis

To overcome the sparsity of single-cell data for both the RNA and ATAC modalities, we also benchmarked the approaches above after forming metacells by SEACells (Persad *et al.* 2023). Specifically, the parameters of SEACells were set to default and each method was run on data from metacells as the experimental units. Collectively, Fig. 1b summarizes the approaches considered in this study.

#### 2.3.3 SCARlink benchmarking

Since SCARlink (Mitra *et al.* 2024) employs regularized Poisson regression on tile-level accessibility data, it produces an FDR value for each gene–genomic tile pair, which is generated by adjusting p-values using the Benjamini–Hochberg method on individual genes. To incorporate SCARlink into the benchmarking pipeline, we use the maximum of 1−FDR across all gene–tile pairs that overlap with the enhancer as the gene-enhancer-level SCARlink association statistic.

### 2.4 Distance adjustment of the gene–enhancer statistics

To acknowledge the impact of the distance between the genes and the enhancers for their interactions, Duren *et al.* (2022) presented scREG, which developed a lower dimensional representation for each cell with a loss function related to the reconstruction of gene expression, chromatin accessibility, and distance-correlated regulatory potential. The recovered distance-correlated regulatory potential for each gene–enhancer pair aggregated over sub-populations is then used as an indicator of the pair’s regulation strength. To compare with scREG, we designed distance adapted versions for all aforementioned methods. For each gene–enhancer pair g−e, the association statistic $s_{ge}$ is adjusted as

$$s_{ge}^{\mathrm{DistAdj}}=(s_{ge}+\epsilon )\cdot e^{-d_{ge}/d_{0}},$$

where ϵ (default 0.05) is to ensure the term is positive, $d_{ge}$ represents the distance between the transcription start site (TSS) of gene g and enhancer e, and the base parameter $d_{0}$ (default 200 kb) reflects the weight decay scale with distance (Duren *et al.* 2022). These distance-weighted statistics tend to boost closer gene–enhancer pairs while downweighting distal pairs as in scREG. In the comparisons that included scREG, these distance adjusted versions of the methods were utilized (Fig. 1d).

### 2.5 Multi-modal single-cell datasets used in this study

We leveraged most recent, high quality multi-modal datasets of cell lines GM12878 (Wu *et al.* 2021), K562 (Xu *et al.* 2022), and human peripheral blood mononuclear cells (PBMCs) (10x Genomics 2021) from the 10X Chromium Single Cell Multiome ATAC + Gene Expression platform for our benchmarking study (Fig. 1c). Two main cell types, namely CD4+ T cells and CD14+ monocytes, were extracted from the PBMC-10X2021 for separate evaluation. Compared to earlier published methods like SNARE-seq (Chen *et al.* 2019) and SHARE-seq (Ma *et al.* 2020), the datasets generated by 10× have higher sequencing depths (Supplementary Table S1c). In addition, for the GM12878-Wu2021, the median numbers of detected genes and peaks across the cells are 3186 and 12 116, respectively, while these numbers for the GM12878 SHARE-seq dataset (Ma *et al.* 2020) are only 528 genes and 806 peaks, respectively. Similarly, 3414 genes and 12 003 peaks were detected in the K562-Xu2022 dataset, while the K562 SHARE-seq dataset (Ma *et al.* 2020) resulted in median of 190 detected genes and 199 detected peaks, respectively. This quality check ensured that our benchmark study is based on the most up-to-date datasets with the highest quality. All candidate enhancer regions (i.e. peaks identified from the scATAC-seq component of the data and are within a window size of 500 kb of genes) were tested for their association with their respective genes. For all the datasets, gene filtering during pre-processing retained genes that (i) are expressed in more than 5% cells; (ii) are within the top 8000 most variable genes according to expression values across the cells; (iii) have at least three *cis*-peaks in single-cell ATAC-seq data, which excludes on average 10 genes. This resulted in 466, 1634, 1752, 4031, 3634 genes, and 33 968, 57 557, 61 937, 66 940, 40 375 candidate enhancers for K562-Xu2022, replicate 1 and 2 in GM12878-Wu2021, CD4+ T cells and CD14+ monocytes in PBMC-10X2021, respectively. The peaks reported in the original data paper were employed for this analysis. Further details on data pre-processing are provided in Supplementary Section S3.

### 2.6 Benchmarking pipeline

Each gene–enhancer interaction inference method was evaluated based on accuracy and reproducibility. Detection accuracy is primarily assessed with AUROC and AUPR. Gold standard gene–enhancer interactions for evaluations were deployed from multiple sources of gene–enhancer interactions that utilized bulk sequencing technologies (Supplementary Table S1, Fig. 1c). Although benchmarking single-cell results against gold standards from bulk experiments is not ideal, the limited availability of single-cell-level gold standards makes this approach commonly used in the studies listed in Supplementary Table S1. To assess reproducibility, we utilized two biological replicates from GM12878-Wu2021. For each gene–enhancer pair $g_{1}-e_{1}$ in replicate 1, if enhancer $e_{1}$ overlaps with enhancer $e_{2}$ in replicate 2, the association statistics for $g_{1}-e_{2}$ in replicate 2 are mapped to $g_{1}-e_{1}$ as the reproduced association statistics. Reproducibility was then quantified by counting the number of gene–enhancer pairs that appear in both the top n association statistics in replicate 1 and the top n reproduced association statistics, with n varying to explore different thresholds.

A significant challenge in benchmarking gene–enhancer interaction detection methods lies in the notion of true negatives. This is because the gold standards primarily operate on detection of interactions and this is prone to errors due to signal-to-noise of the bulk datasets and/or employed technologies. Gold standard datasets derived from high-throughput chromatin conformation capture assays (Bhattacharyya *et al.* 2019) and their variations report physical 3D chromatin contacts and are prone to missing interactions due to sequencing depth limitations, limited numbers of replicates, or other technical limitations. Other gold standard datasets such as FANTOM5-Andersson2014 (Andersson *et al.* 2014) report gene–enhancer pairs based on their cap analysis of gene expression (CAGE) correlation across a diverse set of human cells. Neither type of gold standard datasets explicitly define negative gene–enhancer pairs, posing difficulties in computing accuracy metrics. To address this issue, we considered all reported/detected gene–enhancer pairs of gold standards as true interactions. In our experiments, a candidate gene–enhancer pair was labeled as a negative interaction if the enhancer (i) was among the set of enhancers that the gold standard dataset harbored and (ii) was inferred to interact with a gene different than the one in the gold standard dataset. This definition accounts for the detection limitations of gold standard datasets by evaluating only the detected enhancers. To ensure comprehensiveness, multiple gold standard datasets from a variety of resources (Supplementary Table S3), including 3D chromosome conformation detection techniques, CRISPRi screens, genome-wide association study (GWAS) databases, and atlases of enhancers (Andersson *et al.* 2014, Javierre *et al.* 2016, Bhattacharyya *et al.* 2019, Li *et al.* 2019, Fulco *et al.* 2019, Gasperini *et al.* 2019, Schraivogel *et al.* 2020, Moore *et al.* 2020, Gao and Qian 2020).

## 3 Results

### 3.1 Gene–enhancer interaction detection from multi-modal single-cell data of human cell lines

**Figure 2.**
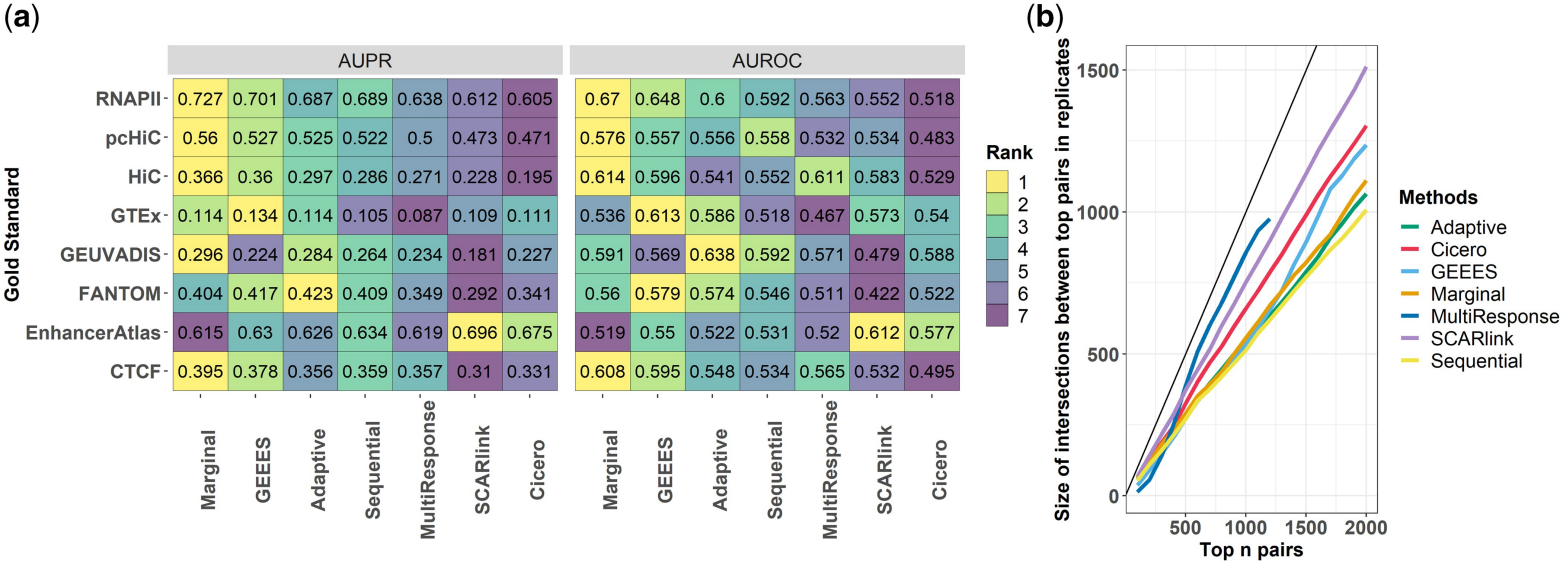
(a) AUPR and AUROC evaluation of GM12878-Wu2021 results based on multiple gold standard datasets. (b) Reproducibility analysis based on the GM12878-Wu2021 dataset. For each method, the number of gene–enhancer interactions that are identified among the top n pairs in both replicates are displayed. *x*-axis: the number of pairs having the highest gene–enhancer interaction scores in each GM12878 replicate. *y*-axis: the number of overlapped pairs identified as top *n* pairs in both replicates (as shown in the *x*-axis). The black line marks the y=x for perfect reproducibility.

We first compared the methods on the analysis of GM12878-Wu2021 and K562-Xu2022 datasets. Here, we describe the results for GM12878-Wu2021 replicate 1 in detail and refer to Supplementary Section S6 for K562-Xu2022 and PBMC-10X2021. Results for GM12878-Wu2021 replicate 1 were evaluated using three sources of gold standards: BENGI-Moore2020 (Moore *et al.* 2020), FANTOM5-Andersson2014 (Andersson *et al.* 2014), and EnhancerAtlas 2.0 (Gao and Qian 2020). Since BENGI-Moore2020 offers gold standards from multiple resources, the benchmarking results based on each of these resources are presented and summarized alongside the other gold standards in Fig. 2a. Overall, the AUROC and AUPR values for all the methods are generally low and markedly similar to each other (within ±0.1). To gain a better understanding of how the association statistics of each method behave, we investigated the distribution of the gene–enhancer association statistics for three groups of gene–enhancer pairs: True—True positive pairs based on pcHiC-BENGI2020 gold standard; False—False positive pairs based on pcHiC-BENGI2020 gold standard; and Not classified—Pairs not labelled by the pcHiC-BENGI2020 gold standard. The distributions of association statistics show a similar pattern when pairs are grouped based on the other resources of BENGI-Moore2020, FANTOM5-Andersson2014 or EnhancerAtlas 2.0. Supplementary Fig. S2b displays the results for GEEES, *Marginal Correlation*, and *Adaptive Regression* (as a representative of the regression methods) where statistics are normalized by subtracting their minimum values in each method and larger values present stronger evidence for association. Wilcoxon one-sided *P*-values for comparing statistics of True and False gene–enhancer pairs classified by gold standards are also provided to measure the differences quantitatively. Encouragingly, the distribution of the association statistics across the True and False classes exhibit an apparent difference for all the methods based on Kolmogorov-Smirnov test (*P*-values < $2.2\times 10^{-16}$). Furthermore, gene–enhancer interactions in the True class, on average, exhibit stronger association statistics. Qualitatively similar results for the other datasets (K562-Xu2022, GM12878-Wu2021: replicate 2, PBMC-10X2021: CD4+ T and CD14+ monocytes) with generally unremarkable differences between the performances of different methods are available in Supplementary Figs S2 and S3.

To quantify reproducibility of the gene–enhancer associations inferred by different methods, we utilized the two biological replicates from GM12878-Wu2021. Figure 2b displays the number of overlapped gene–enhancer pairs that are ranked among the top n pairs of the both sets of interactions identified from the individual replicates. To improve this display, for each method, the gene–enhancer interactions inferred to have exactly zero association statistics are excluded. This comparison yields *multi-response regression*, SCARlink and cicero as the top 3 with the best reproducibility. Notably, both *multi-response regression* and cicero capitalize the relationship between promoter accessibility and enhancer accessibility. This suggests that incorporating the promoter–enhancer accessibility relationship may enhance the robustness of gene–enhancer identification.

### 3.2 Limited effect of aggregating single cells into metacells

Next, we coupled each method with metacell construction by SEACells (Persad *et al.* 2023) to assess whether reducing overall sparsity for the expression and accessibility measurements of the cells benefits individual methods. This resulted in a total of 13–111 metacells across the datasets. We then applied the same benchmarking framework to evaluate the inferred gene–enhancer interactions after metacell construction. Perhaps not so surprisingly, we first observed that GEEES performed markedly worse than before in all datasets (Supplementary Figs S5 and S6), highlighting the single-cell specific nature of GEEES. GEEES operates on a small neighborhood of each single cell to capture both the linear and nonlinear interactions; however, the accessibility distribution within each metacell neighborhood deviates from the Binomial assumption when single cells are aggregated into metacells (Supplementary Section S9).

The performances of the remaining methods appear to be markedly similar to those observed when applied to single-cell level data, both in terms of the values of the evaluation metrics and the ranking among the methods. These results elude to the limited effect of metacell strategy for inferring gene–enhancer interactions. It is worth noting that the poor performance of methods utilizing metacells may be attributed to the relatively small sample sizes of generated metacells, which highlights a trade-off between sample size and sparsity in the context of using multi-modal single-cell data for gene–enhancer regulatory pair detection. Interestingly, this is similar to the limited effect of metacells on inferring co-expression networks from population-scale scRNA-seq data (Lu and Keleş 2023).

### 3.3 Distance adjustment increases performance across methods and strengthens GEEES effectiveness

Next, inspired by the explicit use of the distance information between the genes and candidate enhancers by scREG (Duren *et al.* 2022), we implemented a distance adjustment post-processing that leverages the distance between the candidate enhancer and the TSS of the gene for all the methods. We observe that distance adjustment yields marked improvement in the evaluation metrics for all the methods in most gold standard datasets (Supplementary Fig. S7a). For example, in the GM12878-Wu2021 replicate 1 dataset, all methods achieve marked improvements in AUROC (23.4% to 38.9%) and AUPR scores (16.5% to 28.5%) on EnhancerAtlas 2.0 validated pairs.

This improvement is related to a strong distance effect present in the validated gene–enhancer pairs of the gold standard datasets, such as EnhancerAtlas 2.0. When comparing the distance distributions of positive and negative gene–enhancer pairs within these gold standard datasets (Supplementary Fig. S7b), it is evident that positive pairs exhibit significantly shorter distances than negative pairs, as confirmed by two-sample Kolmogorov–Smirnov test *P*-values $<10^{-8}$. This trend holds true across all gold standard datasets, except for HiChIP-Bhattacharyya2019, HiC-BENGI2020 and FANTOM5-Andersson2014. Analysis of these two specific 3D chromatin interaction datasets (HiChIP-Bhattacharyya2019 and HiC-BENGI2020) included a distance-related bias correction to remove the distance bias inherent to the measurements from proximity ligation assays.

**Figure 3.**
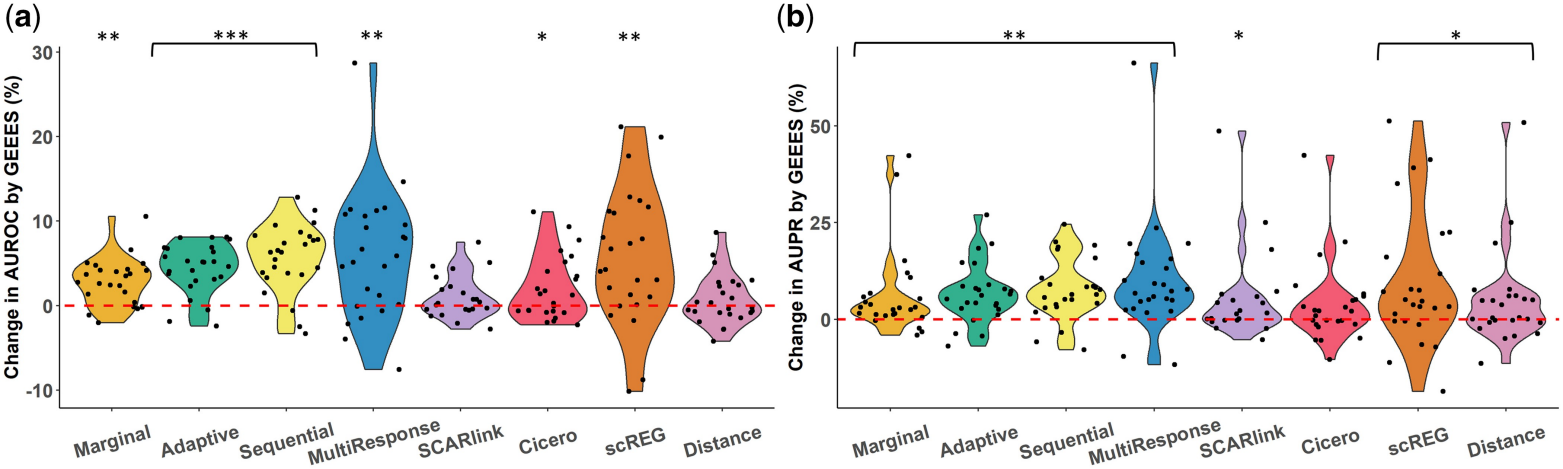
(a) Percentage changes in AUROC by GEEES compared to other methods computed as: $\frac{\text{AUROC}(\text{GEEES})-\text{AUROC}(\text{Method on x-axis})}{\text{AUROC}(\text{Method on x-axis})}\times 100$ among all gold standard datasets. All methods are adjusted by distance. One-sided Wilcoxon *P*-values for testing whether the changes in AUROC by GEEES are positive are reported as ***: *P* < 10^−5^, $**:p\in [10^{-5},10^{-3})$, $*:p\in [10^{-3},0.05)$. (b) Percentage changes in AUPR by GEEES compared to other methods computed and labeled the same as (a) for AUPR.

Given the uniform improvement achieved for all methods by the distance adjustment, we further compared the performances of all the distance-adjusted methods including scREG with the benchmarking framework. Figure 3 compares individual methods to GEEES and Supplementary Fig. S9 presents their absolute performances. We observed that GEEES consistently outperforms the other benchmarked methods across all test datasets on most gold standard datasets, in terms of both AUROC and AUPR relatively (Wilcoxon one-sided *P*-values <0.05 for all comparisons). It is worth noting that while GEEES exhibits marked improvements over other methods, its absolute improvement is not remarkable, given that all these methods are still just on par with the “Distance” baseline (Fig. 3) which ranks enhancers for a given gene solely based on their linear distance from TSS (Supplementary Fig. S9). This is with the exception that GEEES yields an average of 3.2% higher AUPR than the distance baseline (Fig. 3b; Wilcoxon one-sided *P*-value <0.05). Collectively, these findings underscore the critical role of considering distance information for accurate detection of gene–enhancer regulatory pairs from single-cell data, which is similar to the observation in Moore *et al.* (2020) on inferring gene–enhancer pairs from bulk data.

#### 3.4 Characteristics of the gold standard datasets

To further scrutinize the markedly similar poor performances of different classes of methods for gene–enhancer interaction inference and the observed distance bias, we investigated characteristics of different gold standard datasets used for benchmarking in Supplementary Section S11. We observed a low agreement among gold standards for each benchmarking dataset (Supplementary Fig. S10), and noted the disagreement between actual interaction signals and gold standard detection (Supplementary Fig. S12). These findings suggest that the evaluation based on the current gold standard is limited and potentially incomplete. Three-dimensional chromatin interaction gold standards, such as HiChIP-Bhattacharyya2019, measure physical interactions. In contrast, correlation analysis based on multi-modal single-cell data infers correlations between gene expression and peak accessibility. This correlation can be confounded by co-expression between genes and does not necessarily imply a physical interaction. On the other hand, for gene–enhancer pairs with physical interactions, gene expression and enhancer accessibility are not necessarily highly correlated due to technical limitations, such as sparsity in multi-modal single-cell data, which may even fail to identify the accessibility of the enhancer. Moreover, in enhancer–promoter pairs exhibiting physical interactions within the 3D chromatin structure, it has been observed that alterations in acetylation (which renders DNA more accessible) at distal enhancers precede changes in gene expression (Reed *et al.* 2022). This sequential progression in enhancer accessibility and gene expression changes might elucidate the discrepancy between correlation-based findings and gold standards rooted in 3D chromatin interactions. This arises from the fact that correlations may not adequately capture temporally ordered associations, especially when the data is collected concurrently. Furthermore, although CRISPRi screen experiments (Gasperini *et al.* 2020) provide more direct measurement of enhancer regulatory effects on target genes, the number of validated gene–enhancer pairs is currently extremely limited and the pairs do not show substantial overlap due to differences in design (Supplementary Fig. S4b). Collectively, these findings suggest the need for better and more comprehensive benchmark gold standard datasets for validating gene–enhancer interactions inferred from single-cell data.

#### 3.5 GEEES facilitates detection of cell sub-populations

**Figure 4.**
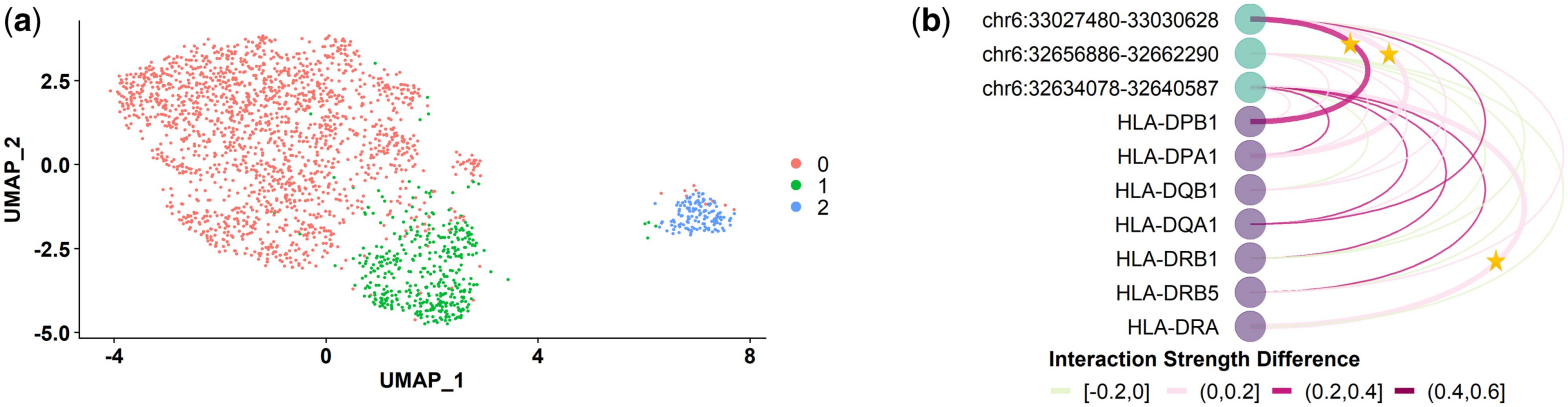
(a) UMAP visualization of CD14+ monocytes using GEEES statistics for all gene–enhancer pairs as features and colored by clustering result. (b) The differences between the normalized GEEES interaction strengths in cluster 0 and cluster 1 from (a) on HLA family genes and related enhancers shown as nodes. For gene *g* and enhancer *e* pair in cluster *i*, the normalized GEEES interaction strength is defined as ${\text{GEEES}}_{N\mathrm{orm}}(\mathrm{gei})=\frac{\text{GEEES}(\mathrm{gei})}{max_{e^{\prime }\in E_{g},i^{\prime }\in \{0,1\}}\text{GEEES}(ge^{\prime }i^{\prime })}$, where GEEES(gei) is the median aggregation of *g*-*e* GEEES statistics among all cluster *i* cells. The edge colors correspond to ${\text{GEEES}}_{N\mathrm{orm}}(ge1)-{\text{GEEES}}_{N\mathrm{orm}}(ge0)$. The gene–enhancer pairs validated by the pcHiC-Javierre2016 are marked by stars on the edges.

Next, we explored how GEEES enables dimension reduction based on summarized gene–enhancer interactions at the cell level. To showcase this analysis, we considered the top 0.1% gene–enhancer interactions identified by GEEES in the CD14+ monocytes dataset, and leveraged their cell-specific GEEES statistics as cell features for the clustering analysis with Seurat package standard pipeline (Hao *et al.* 2021) (normalization, dimension reduction and Louvain clustering with default parameters). We further restricted these features in a cell to be zero when the gene was not expressed and/or the enhancer was not accessible. The UMAP visualization of the clustering result on 2314 cells is displayed in Fig. 4a. An investigation of the relationship between sequencing depths of the cells and the clustering result (Supplementary Fig. S13) suggested that cluster 2 could be driven by sequencing depth differences. In contrast, clusters 0 and 1 appeared to be mainly driven by differences in marker gene–enhancer interactions, eluding to potential gene regulatory patterns within the same cell type. In this context, marker gene–enhancer interactions are identified utilizing the FindMarkers function within the Seurat package, incorporating GEEES statistics as cell features for comparison between clusters 0 and 1.

Specifically, we identified a group of HLA genes (HLA-DPB1, HLA-DPA1, HLA-DQB1, HLA-DQA1, HLA-DRB1, HLA-DRB5 and HLA-DRA) and enhancers at chr6:32 634 078–32 640 587, chr6:32 656 886–32 662 290 and chr6:33 027 480–33 030 628 associating more strongly in cluster 1. Three of these gene–enhancer pairs (marked with stars in Fig. 4b and Supplementary Fig. S14: HLA-DRA-chr6:32 634 078–32 640 587, HLA-DPA1-chr6:33 027 480–33 030 628, and HLA-DPB1-chr6:33 027 480–33 030 628) are validated by pcHiC-Javierre2016 in CD14+ monocytes. All of these pairs exhibit stronger interaction strength in cluster 1. This set of HLA genes belongs to the human leukocyte antigen (HLA) complex, which aids the immune system in distinguishing the body’s own proteins from those produced by foreign invaders such as viruses and bacteria (Choo 2007). This discovery underscores the potential of GEEES cell-specific statistics in examining the heterogeneity of regulatory patterns at the cellular level. Differentially associating gene–enhancer pairs within the same cell type may also contribute to the disparities between gene–enhancer detection results based on multi-modal single-cell data and the gold standards derived from bulk data.

## 4 Discussion

In this study, we proposed and explored different classes of statistical methods for identifying gene–enhancer interactions from multi-modal single-cell data. These included GEEES, a cell-specific method that infers such interactions at the single-cell level, *marginal correlation* analysis (Ma *et al.* 2020), cicero (Pliner *et al.* 2018), multivariate and/or multiple response regression methods [*Adaptive*, *Sequential*, *Multi-Response*, SCARlink (Mitra *et al.* 2024)], and a distance-based method scREG (Duren *et al.* 2022). Benchmarking experiments on high quality multi-modal single-cell data with a wide variety of gold standard datasets yielded uniformly poor performances for all the methods. The comparable performance between cicero and the other methods benchmarked suggests that the current approaches do not fully leverage the interplay between chromatin accessibility and gene expression. Coupling each method with a metacell approach to alleviate apparent sparsity of the single-cell data modalities did not lead to better performances. Instead, we observed that a simple distance adjustment markedly improved the performances of all the methods and elicited GEEES as the best performing. Despite this, a simple distance-based baseline approach that gave priority to the closest enhancers remained as competitive with these methods. Investigating the gold standard datasets in more depth, we attributed the poor performances of the methods to the lack of concordance between gold standard datasets and the strong distance effect. The correlation-based nature of the methods, coupled with the potential heterogeneity of regulatory patterns within the same cell type, further contributes to the disparity between interaction results obtained from single-cell data and bulk 3D chromatin interaction gold standards. This discrepancy is influenced by factors such as confounding gene co-expression, technical limitations like sparsity in multi-modal single-cell data, and the sequential nature of gene–enhancer interaction and gene expression in gene regulation. Collectively, these findings highlight the need to generate better benchmark datasets for validating single-cell inferred gene–enhancer interactions.

## Supplementary Material

btae638_Supplementary_Data

## Data Availability

The data underlying this article are available through Gene Expression Omnibus (GEO) at https://www.ncbi.nlm.nih.gov/geo/ with accession number GSE166797, European Molecular Biology Laboratory's European Bioinformatics Insitute (EMBL-EBI) at https://www.ebi.ac.uk/ with accession number E-MTAB-11264, and 10X Genomics at https://www.10xgenomics.com/resources/datasets/pbmc-from-a-healthy-donor-granulocytes-removed-through-cell-sorting-10-k-1-standard-2-0-0.
