# Supplementary material for: GEEES: inferring cell-specific gene–enhancer interactions from multi-modal single-cell data
Source: Bioinformatics. 2024 Oct 28;40(11):btae638. doi: 10.1093/bioinformatics/btae638 (PMC11549018; doi:10.1093/bioinformatics/btae638)
Supplement: btae638_Supplementary_Data [file btae638_supplementary_data.pdf]

Supplementary Materials for "GEEES: Inferring Cell-specific Gene Enhancer Interactions from Multi-modal Single Cell Data"

Shuyang Chen[1] and Sündüz Keleş[1,2,*]

[1]Department of Statistics, University of Wisconsin-Madison, Madison, WI, 53706, USA,
[2]Department of Biostatistics and Medical Informatics, University of Wisconsin-Madison, Madison, 53706, WI, USA.
Corresponding author: *keles@wisc.edu

1 Summary of methods for identifying gene-enhancer interactions from single cell ATAC-seq and RNA-seq data

Method	Statistics addressing gene/promoter -enhancer interaction	Modalities paired or integrated	Univariate#	Error control	Datasets	Gold standard used for validation	Benchmarked in this study
Marginal Method (Ma *et al.*, 2020)	P-value from testing Pearson correlation against non-interacting peaks with GC correction	Paired	Yes	FDR	Mouse skin, GM12878	HiChIP	Yes
scREG (Duren *et al.*, 2022)	Recovered regulatory potential from dimension reduction	Paired	Yes*	Not specified, top 10,000 returned	PBMC	eQTL, HiC	Yes
TRIPOD (Jiang *et al.*, 2022)	P-values of trio-relationships (enhancer-TF-gene) from nonparametric test	Paired	Yes	Bonferroni	PBMC, Mouse skin	FANTOM5, 4DGenome, EnhancerAtlas, GTEx	No, since TRIPOD targets for enhancer-TF-gene trios and does not provide a method to further infer enhancer-gene interactions interested in this study
Cicero (Pliner *et al.*, 2018)	Coaccessibility scores assigned to promoter-peak pairs using Graphical LASSO	Single-cell ATAC-seq	No	Not specified	Human skeletal muscle myoblasts, GM12878, Immortalized human myoblasts	ChIA-PET, HiC	Yes
FigR (Kartha *et al.*, 2022)	Marginal method is applied in a step for inferring gene-enhancer pairs	Integrated /Paired	Yes	FDR	PBMC, GM12878	ChIP-seq	Yes, included as the marginal method
snapATAC (Fang *et al.*, 2021)	Logistic regression between each gene-enhancer pair is applied to the generated pseudo multi-omics data	Integrated	Yes	not specified	PBMC	{cis}-eQTL	Yes, included as the marginal method
SCARlink (Mitra *et al.*, 2024)	The regression coefficient of regularized Poisson regression predict gene expression from tile-level data	Paired	No	Smoothed p-value by locally adaptive weighting and screening	PBMC, BMMC, Pituitary gland, Mouse skin and Human cortex	PCHi-C and fine mapped eQTL	Yes

Table 1: Summary of state-of-the-art gene-enhancer identification methods which use single-cell ATAC-seq and/or single-cell RNA-seq data. Modality "Paired" refers to data from a multi-modal single cell assay whereas "integrated" refers to separately profiled and computationally integrated single cell data. * The regulatory potential in `scREG` is computed for each gene-enhancer pair independently while the dimension reduction step leverages all pairs simultaneously. #: Are the gene-enhancer interactions identified one at a time?, i.e., as opposed to a multivariate model.

2 Details on stability selection

For regression-based methods (*adaptive*, *sequential*, and *multi-response*), a range of penalty parameters (PP) is employed, spanning from $0.01L$ to L, where $L = \max_{e \in E_g}(\sum_{c=1}^{C} A_{ce} Y_{cg})$. Here, Y_{cg} represents the expression of gene g in cell c, A_{ce} stands for the accessibility of enhancer e in cell c, and E_g denotes the set of candidate enhancers for gene g.

In the case of *multi-response*, all pairs of values in PP are utilized as penalty parameters for ℓ-1 and ℓ-2 regularization, separately. By setting PFER = 0.8, $q = 3$, and sampling.type = MB, stability selection provides one selection rate for each gene-enhancer pair under each penalty setting. The highest selection rate among all penalty settings is employed for quantification of the gene-enhancer associations.

3 Details of datasets used for benchmarking

Method	Samples	RNA sequencing depth	ATAC sequencing depth
SNARE-seq	Adult mouse brain	1,546.08	2,634.22
(Chen *et al.*, 2019)	P0 mouse brain	402.34	2,703.00
SHARE-seq	K562	254.31	240.92
(Ma *et al.*, 2020)	GM12878	1,143.20	1,137.35
	K562	10,596.68	28,141.27
10X Single-Cell Multiome	GM12878 replicate 1	13,179.14	38,528.83
(De Rop *et al.*, 2023)	GM12878 replicate 2	10,090.65	35,058.85
	PBMC	4,359.17	22,006.60

Table 2: Comparison of sequencing depths from different multi-modal single cell scRNA-seq and scATAC-seq profiling methods. The sequencing depths for the scRNA-seq and scATAC-seq components are determined based on the average total UMI counts and the average total number of fragments across cells, respectively.

Table 2 provides the average sequencing depths of the datasets used in benchmarking. For K562-Xu2022, 8,240 retained cells have total accessibility counts between 1,000 and 100,000 and total gene expression counts between 2,000 and 30,000. For GM12878-Wu2021 replicate 1, 3,258 retained cells have total accessibility counts between 100 and 500,000 and total gene expression counts between 1,000 and 100,000. For GM12878-Wu2021 replicate 2, 3,416 cells retained cells have total accessibility counts between 500 and 500,000 and total gene expression counts between 500 and 100,000. PBMC-10X2021 is preprocessed in the same manner as described in (Jiang *et al.*, 2022), resulting in 970 and 2,314 cells for CD4+ T cells and CD14+ monocytes, respectively.

Single-cell RNA-seq data is normalized using `sctransform` (Hafemeister and Satija, 2019), while single-cell ATAC-seq data is normalized using TFIDF for all datasets.

4 Details of the gold standard datasets used for benchmarking

Gold standard	Integration	3D chromatin interaction	Correlation based
BENGI (Moore *et al.*, 2020)	✓ ChIA-PET, Hi-C and eQTLs	×	×
EnhancerAtlas (Gao and Qian, 2020)	✓ H3K4me1/H3K27ac, DNase-seq/ATAC-seq, P300, POLR2A, CAGE, ChIA-PET, GRO-seq, STARR-seq and MPRA	×	×
pcHiC (Javierre *et al.*, 2016)	×	✓	×
HiChIP (Bhattacharyya *et al.*, 2019)	×	✓	×
ChIA-PET (Li *et al.*, 2019)	×	✓	×
FANTOM5* (Andersson *et al.*, 2014)	×	×	✓
CRISPRi screen (Fulco *et al.*, 2019; Gasperini *et al.*, 2019; Schraivogel *et al.*, 2020)	×	×	×

Table 3: Data sources of gene-enhancer interactions in each gold standard dataset. *: Using Cap Analysis of Gene Expression (CAGE).

5 Supplementary overall summary table of benchmark results including K562-Xu2022

		AUROC						AUPR					
			PBMC-10X2021		GM12878-Wu2021		K562-		PBMC-10X2021		GM12878-Wu2021		K562-
		Overall	CD14+	CD4+	rep1	rep2	Xu2022	Overall	CD14+	CD4+	rep1	rep2	Xu2022
Original	Marginal	0.565	0.516	0.458	0.664	0.623	0.333	0.544	0.521	0.509	0.591	0.556	0.59
	GEEES	0.554	0.521	0.46	0.63	0.604	0.329	0.537	0.525	0.503	0.576	0.545	0.606
	Adaptive	0.551	0.493	0.452	0.646	0.612	0.332	0.531	0.495	0.504	0.574	0.551	0.564
	Sequential	0.55	0.495	0.45	0.648	0.608	0.328	0.532	0.5	0.503	0.579	0.545	0.571
	Multi-response	0.542	0.514	0.45	0.61	0.596	0.311	0.524	0.512	0.5	0.545	0.538	0.543
	Cicero	0.549	0.528	0.461	0.606	0.6	0.351	0.521	0.514	0.504	0.529	0.538	0.552
Metacell	Marginal	0.507	0.477	0.405	0.56	0.584	0.291	0.519	0.513	0.505	0.517	0.54	0.551
	GEEES	0.465	0.447	0.401	0.502	0.511	0.216	0.471	0.475	0.499	0.451	0.458	0.441
	Adaptive	0.505	0.466	0.399	0.566	0.588	0.317	0.512	0.494	0.491	0.522	0.541	0.561
	Sequential	0.501	0.464	0.401	0.563	0.575	0.323	0.513	0.498	0.499	0.524	0.532	0.574
	Multi-response	0.51	0.48	0.395	0.573	0.592	0.337	0.524	0.509	0.493	0.541	0.554	0.576
Distance Adjusted	Marginal	0.679	0.602	0.546	0.795	0.774	0.527	0.678	0.603	0.601	0.767	0.741	0.713
	GEEES	0.691	0.61	0.555	0.806	0.794	0.548	0.691	0.611	0.605	0.78	0.767	0.743
	Adaptive	0.652	0.573	0.52	0.772	0.744	0.506	0.654	0.582	0.577	0.743	0.715	0.736
	Sequential	0.655	0.576	0.531	0.772	0.742	0.496	0.652	0.584	0.578	0.738	0.707	0.734
	Multi-response	0.654	0.595	0.542	0.747	0.732	0.525	0.658	0.603	0.604	0.723	0.702	0.748
	Cicero	0.682	0.61	0.55	0.787	0.782	0.56	0.683	0.612	0.605	0.758	0.755	0.735
	scREG	0.679	0.664	0.537	0.762	0.754	0.441	0.676	0.669	0.6	0.716	0.718	0.692

Rank: 1 2 3 4 5 6 7 8 9 10 11 12 13 14 15 16 17 18

Figure 1: Supplementary to Fig. 1d with K562-Xu2022 benchmark results included while excluding `SCARlink` due to the lack of the fragment file. Overall summary of the benchmarking experiments, both for individual datasets and in aggregate (Overall). The rankings of methods in each dataset are displayed along the columns with the numerical performance metrics. The numerical performance metrics are calculated based on aggregated gold standard where a gene-enhancer pair is true positive if it is validated by any gold standard for that dataset.

6 Supplementary results without distance adjustment

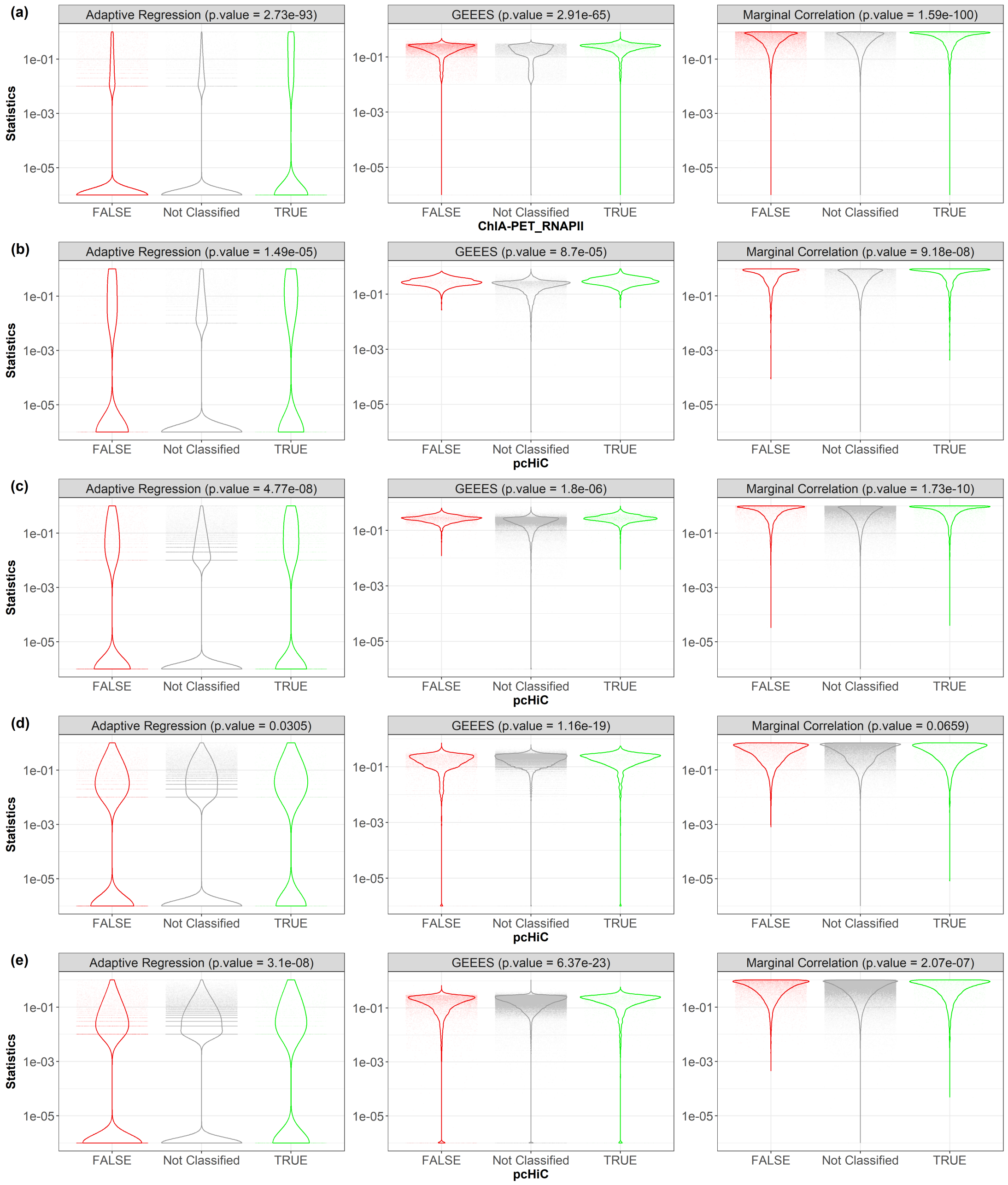

Figure 2: The distributions of the association statistics for (a) K562-Xu2022, (b) GM12878-Wu2021 replicate 1, (c) GM12878-Wu2021 replicate 2, (d) CD4+ T cells and (e) CD14+ monocytes in PBMC-10X2021 dataset from different methods for gene-enhancer pairs that are labelled as True, False, or Not Classified by ChIA-PET-Li2019, pcHiC-Javierre2016, or FANTOM5-Andersson2014 gold standard datasets. A Wilcoxon one-sided p-value (adjusted for multiplicity within each method across all 5 datasets with Bonferroni correction) for testing the difference between association statistics of True and False gene-enhancer pairs for each method is shown in subplot titles.

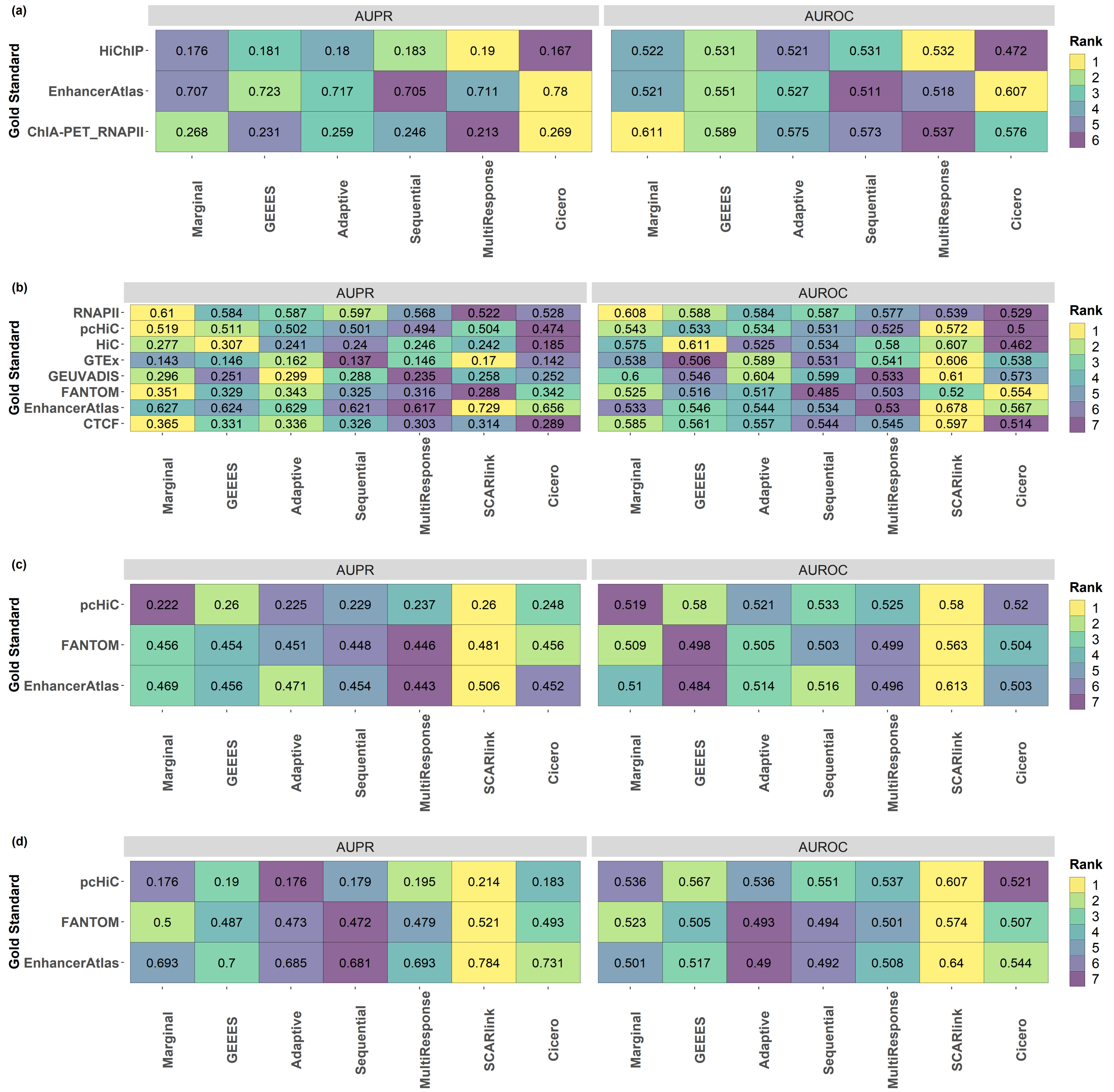

Figure 3: AUPR and AUROC evaluations of (a) K562-Xu2022, (b) GM12878-Wu2021 replicate 2, (c) CD4+ T cells and (d) CD14+ monocytes in PBMC-10X2021 results based on multiple individual gold standard datasets.

7 Supplementary CRISPRi screen evaluation

CRISPRi screen datasets (CRISPRi-screen-Schraivogel2020, CRISPRi-screen-Gasperini2019 and CRISPRi-screen-Fulco2019) processed by Hecker *et al.* (2023) are used to evaluate gene-enhancer pairs inferred from K562-Xu2022. Due to the limited number of gene-enhancer pairs validated in these datasets (8 in CRISPRi-screen-Schraivogel2020, 55 in CRISPRi-screen-Gasperini2019, and 14 in CRISPRi-screen-Fulco2019), we did two adjustments in the evaluation: (i) Three CRISPRi-screen gold standards are aggregated where a gene-enhancer pair is true if it is validated by at least one CRISPRi-screen gold standard; (ii) The resulting scores for all methods are transformed according to their rank as `ceiling(rank(score)/length(score)*10)`, i.e. 10 if the pair has the top 10% highest score, 9 if the score of the pair is ranked between top 10% and top 20%,...,etc. Higher score presents stronger evidence for association.

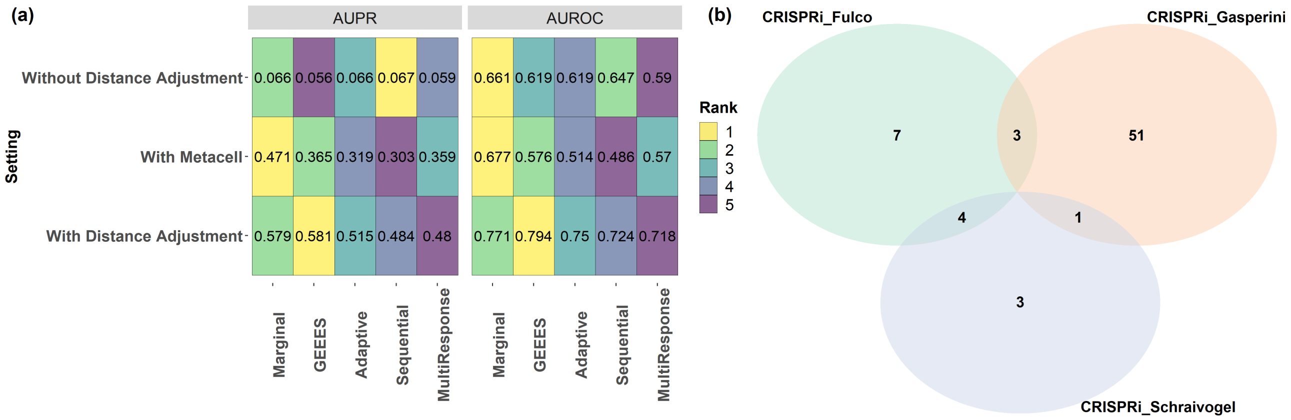

Figure 4: (a) AUPR and AUROC evaluation results of K562-Xu2022 for methods without distance adjustment, with metacells, and with distance adjustment on the aggregated CRISPRi screen gold standard dataset. (b) Comparison of the validated gene-enhancer pairs among different CRISPRi screen gold standard datasets for K562-Xu2022.

8 Supplementary metacell results

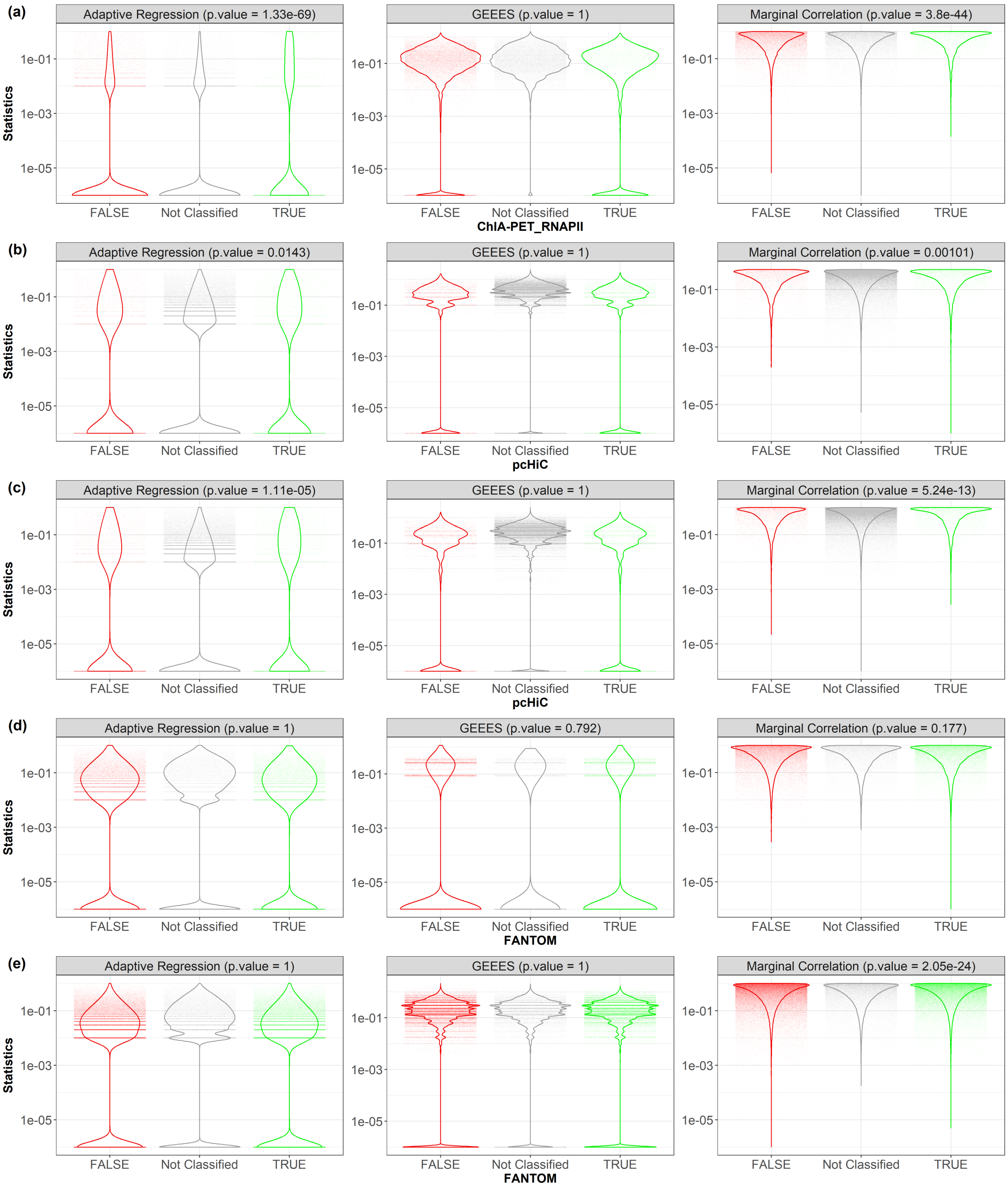

Figure 5: The distributions of the association statistics for (a) K562-Xu2022, (b) GM12878-Wu2021 replicate 1, (c) GM12878-Wu2021 replicate 2, (d) CD4+ T cells and (e) CD14+ monocytes in PBMC-10X2021 with metacells from different methods for gene-enhancer pairs that are labelled as True, False, or Not Classified by ChIA-PET-Li2019, pcHiC-BENGI2020 or FANTOM5-Andersson2014 gold standard datasets. A Wilcoxon one-sided p-value (adjusted for multiplicity within each method across all 5 datasets with Bonferroni correction) for testing the difference between association statistics of True and False gene-enhancer pairs for each method is shown in subplot titles.

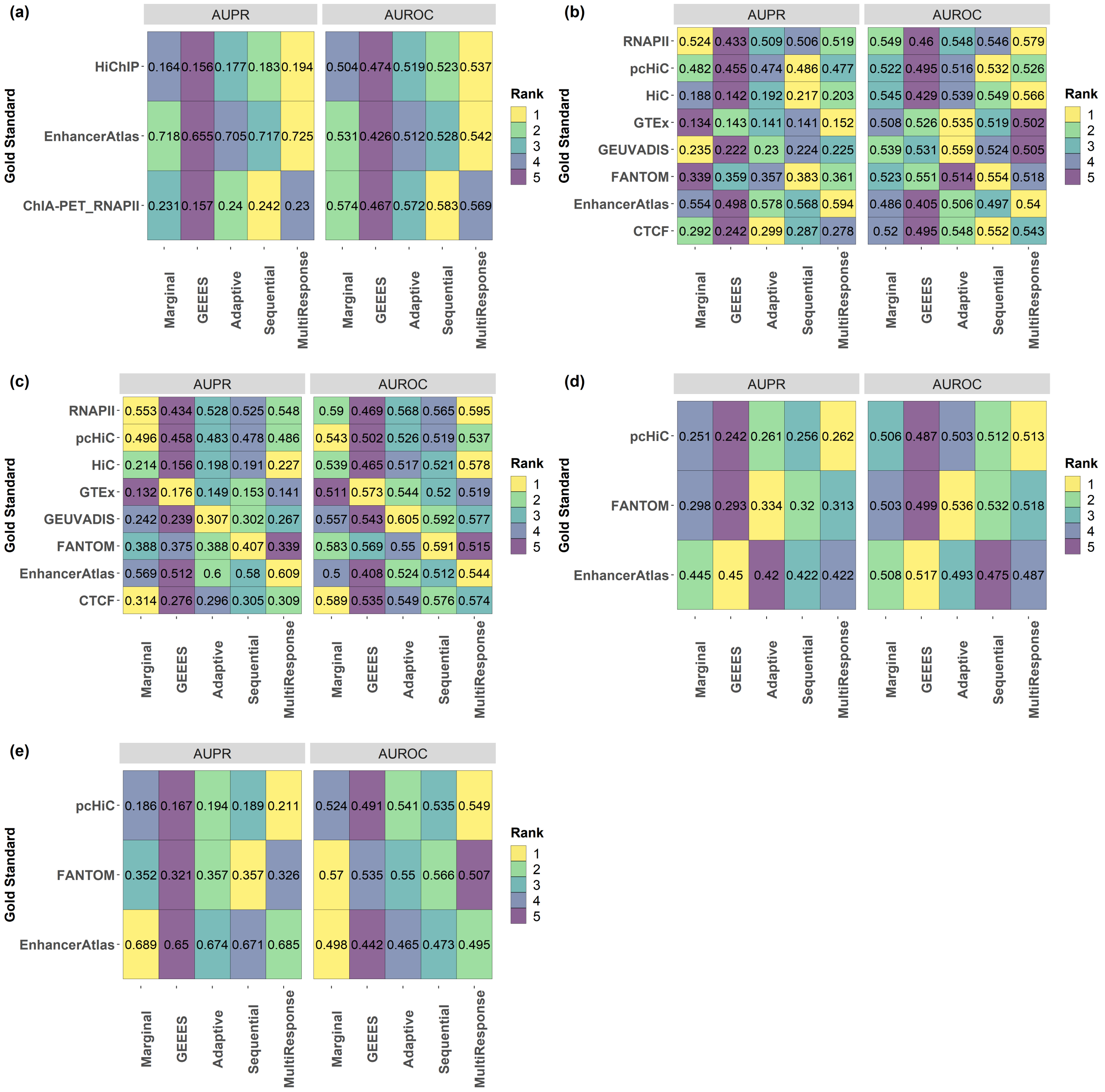

Figure 6: AUPR and AUROC evaluation of (a) K562-Xu2022, (b) GM12878-Wu2021 replicate 1, (c) GM12878-Wu2021 replicate 2, (d) CD4+ T cells and (e) CD14+ monocytes in PBMC-10X2021 with metacells based on multiple individual gold standard datasets.

9 Binomial assumption violation in metacells

In GEEES, accessibility of enhancer e in a single cell c is assumed to have a Bernoulli distribution with $P(A_{ce} = 1) = \pi_e$, where A_{ce} represents the accessibility of e in c. Let's consider the formation of a metacell m comprised of a set of single cells $\mathbb{S}_m$ where $|\mathbb{S}_m| = NC_m$. The accessibility of e in m is defined as $AM_{me} = \mathbb{1}(\sum_{i \in \mathbb{S}_m} A_{ie} > 0)$. Since A_{ie} are independently distributed, $P(AM_{me} = 0) = P(\cap_{i \in \mathbb{S}_m} \{A_{ie} = 0\}) = \Pi_{i \in \mathbb{S}_m} P(A_{ie} = 0) = (1 - \pi_e)^{NC_m}$. Consequently, AM_{me} follows a Bernoulli distribution with $P(AM_{me} = 1) = 1 - (1 - \pi_e)^{NC_m}$.

When applying GEEES to metacell m, a neighborhood $\mathbb{N}_{mg}$ consisting of n_{mg} metacells on gene g is constructed. Defining N_{mge} as the number of neighborhood metacells accessible at enhancer e, we have $N_{mge} = \sum_{i \in \mathbb{N}_{mg}} AM_{ie}$, which is a sum of independent Bernoulli variables with varying probabilities of success due to the different values of $NC_i, i \in \mathbb{N}_{mg}$ among metacells. Therefore, N_{mge} follows a Poisson Binomial distribution rather than a Binomial distribution.

10 Results with distance-adjustment

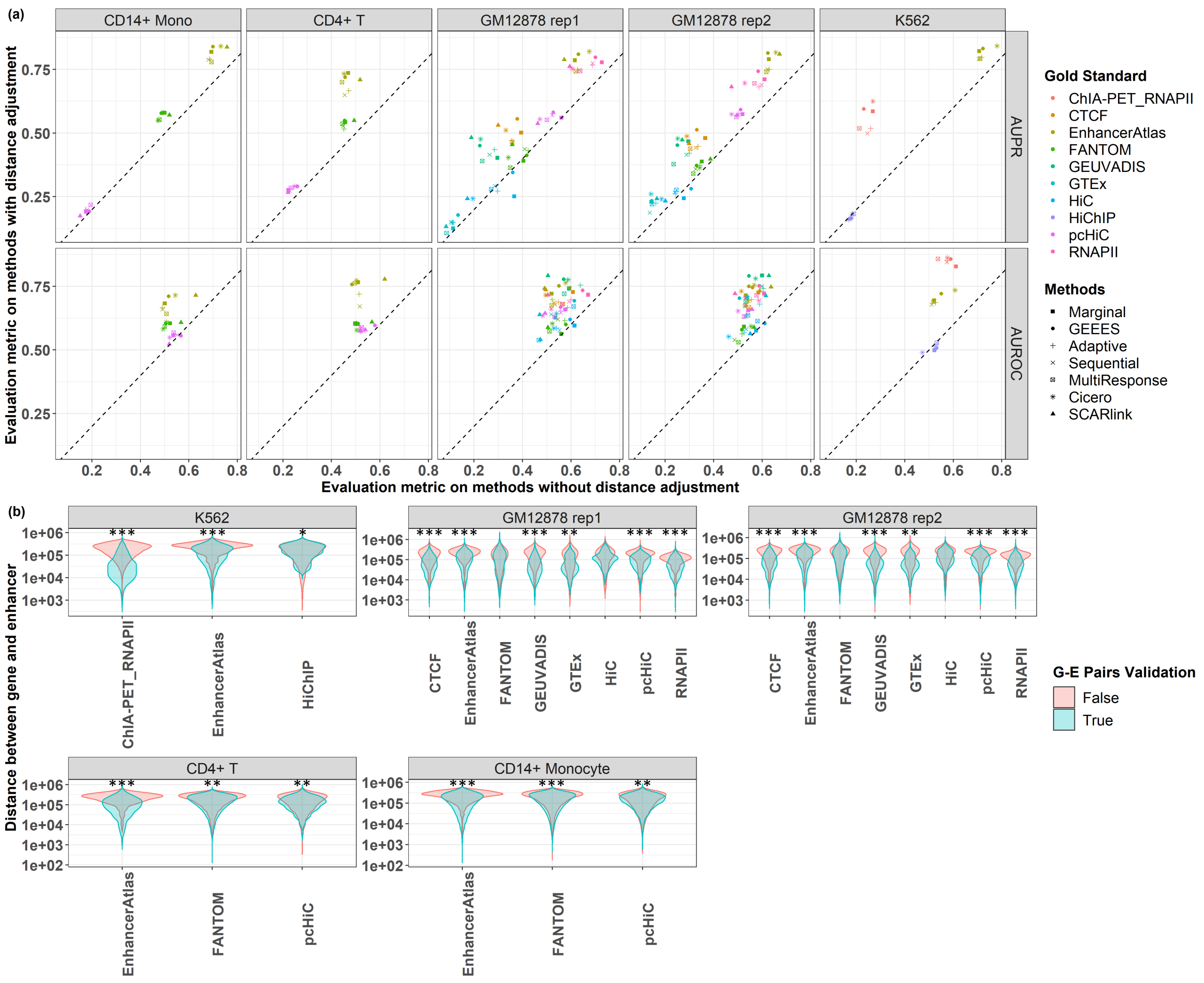

Figure 7: (a) Comparison of performances of methods with (y-axis) and without (x-axis) distance adjustment on all datasets and gold standards. (b) Distribution of distances between genes and enhancers in individual pairs that are labelled True or False based on all gold standard datasets. Two-sample Kolmogorov-Smirnov test p-values from testing whether distances of True pairs and False pairs have equivalent distributions are reported as $*** : p < 10^{-12}$, $** : p \in [10^{-12}, 10^{-8})$, $* : p \in [10^{-8}, 10^{-3})$. Notably, the gold standard datasets derived from pcHiC for GM12878-Wu2021 and PBMC-10X2021 are from different resources. pcHiC for GM12878-Wu2021 is from BENGI (Moore *et al.*, 2020) while pcHiC for PBMC-10X2021 is from Javierre *et al.* (2016) which employed distance bias correction for inferring loops.

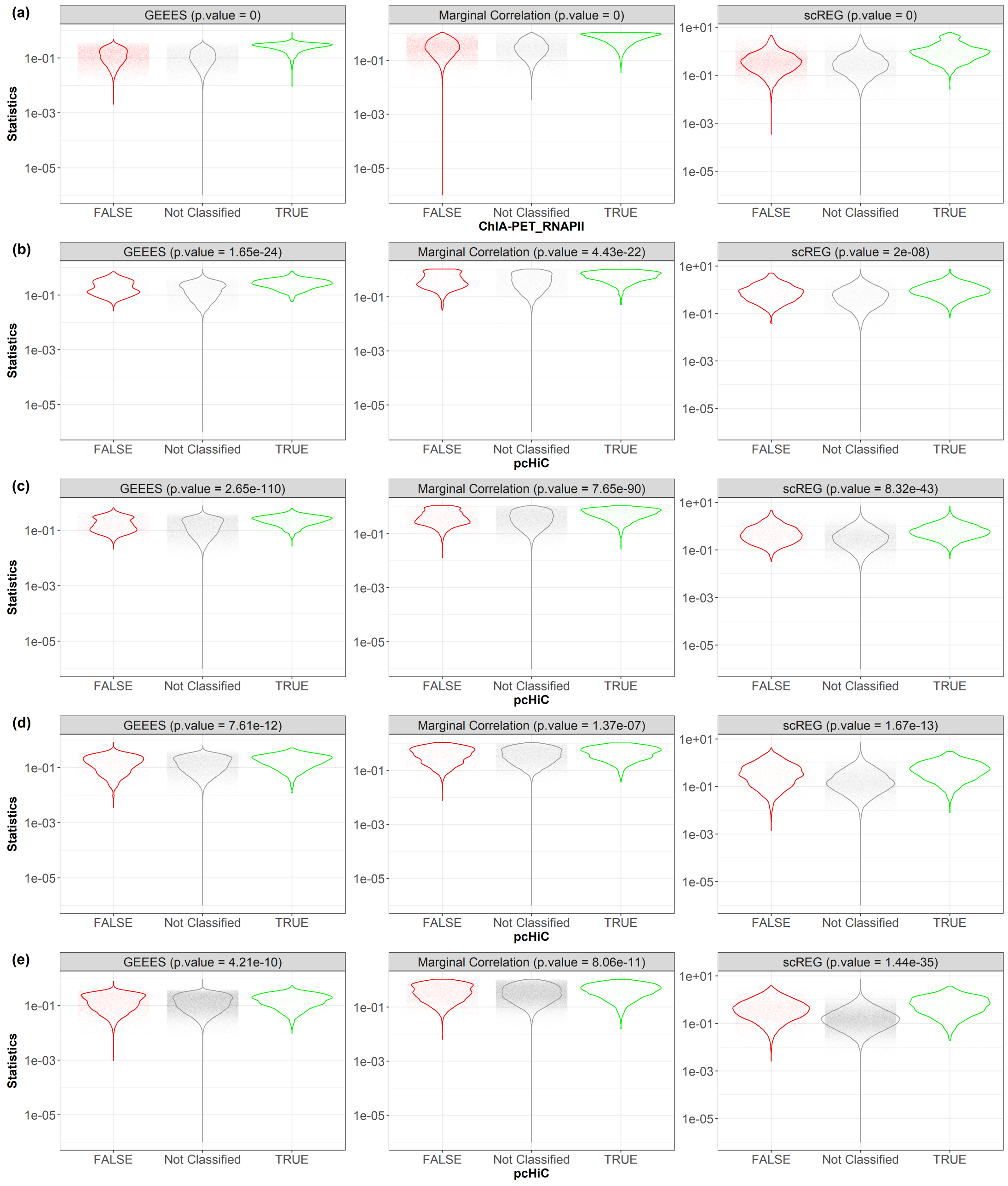

Figure 8: The distributions of the association statistics for (a) K562-Xu2022, (b) GM12878-Wu2021 replicate 1, (c) GM12878-Wu2021 replicate 2, (d) CD4+ T cells and (e) CD14+ monocytes in PBMC-10X2021 with distance adjustment for gene-enhancer pairs that are labelled as True, False, or Not Classified by ChIA-PET-Li2019, pcHiC-BENGI2020, or FANTOM5-Andersson2014 gold standard datasets. A Wilcoxon one-sided p-value (adjusted for multiplicity within each method across all 5 datasets with Bonferroni correction) for testing the difference between association statistics of True and False gene-enhancer pairs for each method is shown in subplot titles.

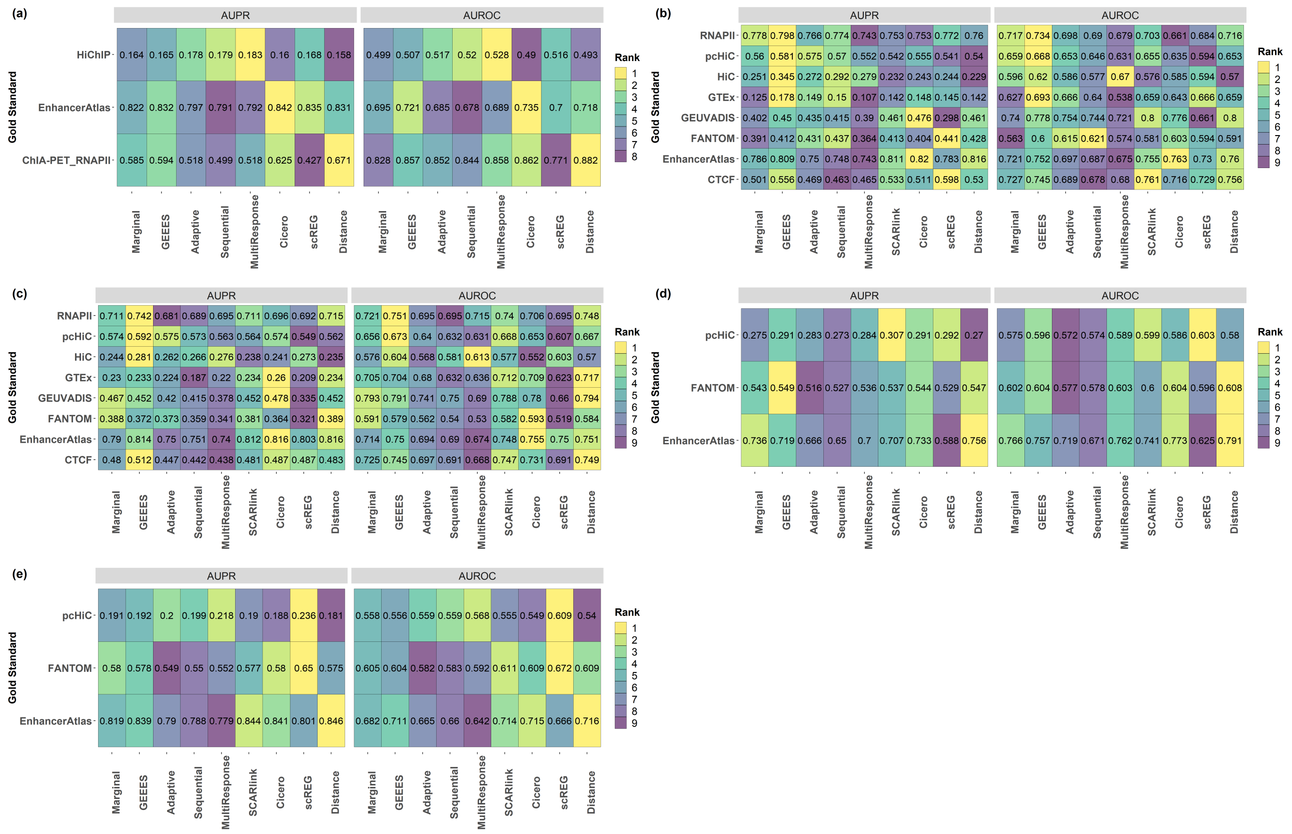

Figure 9: AUPR and AUROC evaluation of (a) K562-Xu2022, (b) GM12878-Wu2021 replicate 1, (c) GM12878-Wu2021 replicate 2, (d) CD4+ T cells and (e) CD14+ monocytes in PBMC-10X2021 with distance adjustment based on multiple gold standard datasets.

11 Investigations of the gold standard datasets

To further scrutinize the markedly similar poor performances of different classes of methods for gene-enhancer interaction inference and the observed distance bias, we compared different gold standard datasets used for each benchmarking dataset in Sup. Fig. 10.

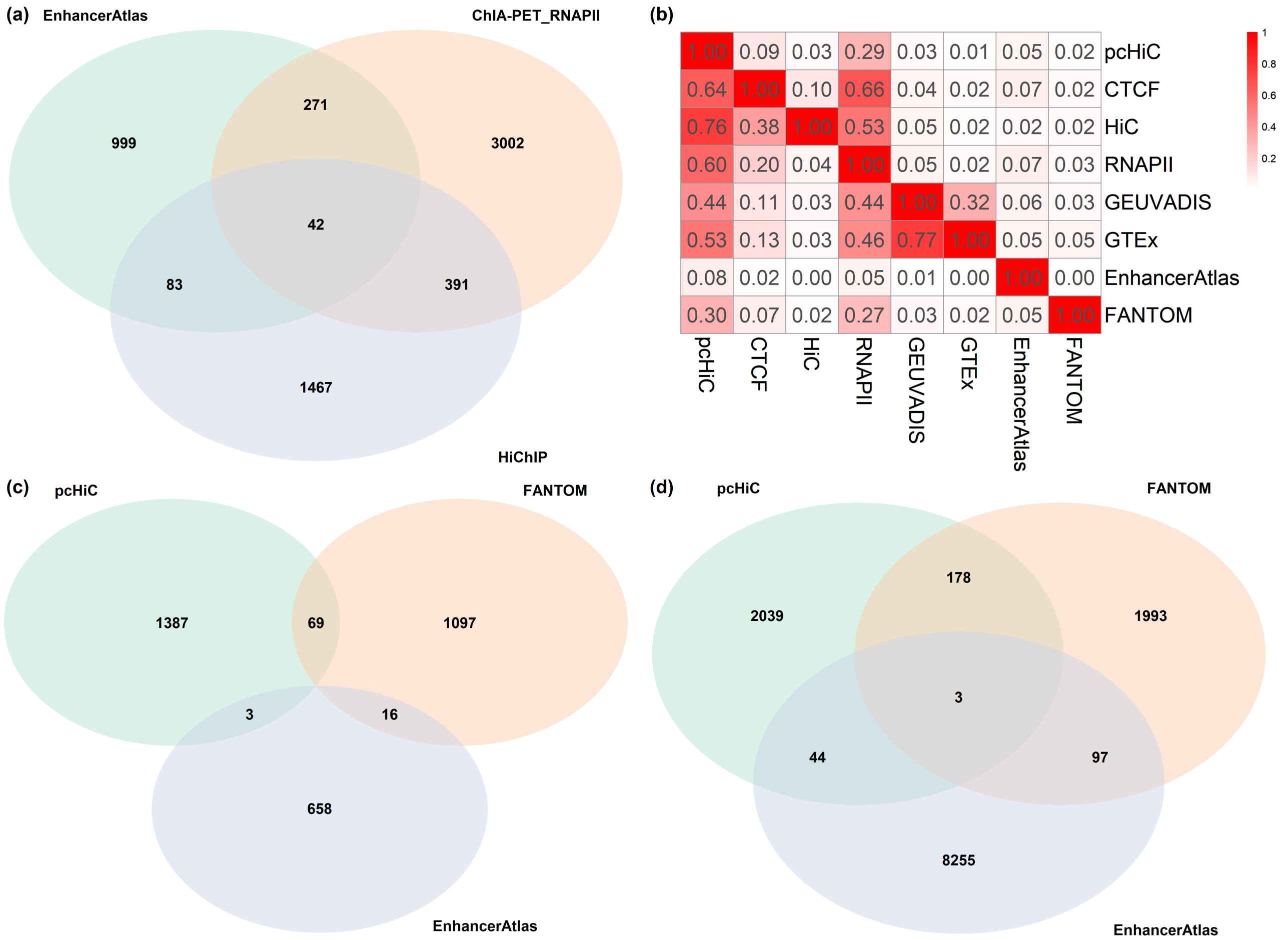

Figure 10: (a) Comparison of the validated gene-enhancer pairs among different gold standards for K562-Xu2022. (b) Each cell in the heatmap depicts the proportion of pairs validated by the gold standard on the row that are also validated by the gold standard on the column for GM12878-Wu2021 gold standards. (c) Comparison of the validated gene-enhancer pairs among different gold standards for the CD4+ T cells in PBMC-10X2021. (d) Comparison of the validated gene-enhancer pairs among different gold standards for the CD14+ monocytes in PBMC-10X2021.

To generate a more comprehensive gold standard dataset for validation, we aggregated the individual gold standard datasets by labeling a pair as true if it is identified in at least one gold standard dataset. We then evaluated all the methods with this aggregated gold standard dataset (AUPR and AUROC are provided in Fig. 1d). Sup. Fig. 11 quantifies precision among the top ranked pairs of each method and yields that none of the methods without distance adjustment can exceed 65% precision.

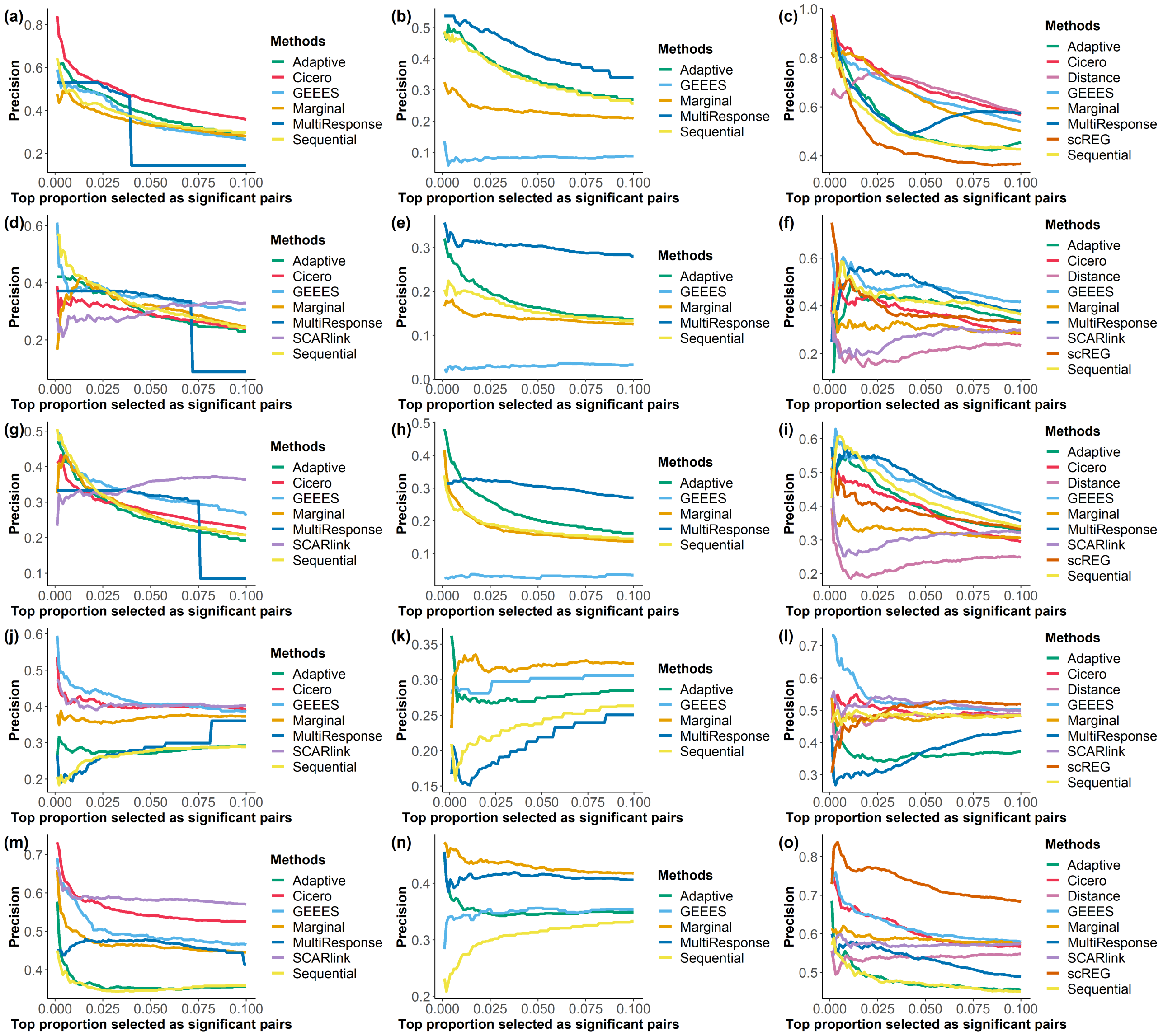

Figure 11: Precision vs. top proportion of gene-enhancer pairs selected as significant pairs for each method using aggregated gold standards for evaluation in K562-Xu2022 ((a) without distance adjustment (b) metacell result, (c) distance adjustment), GM12878-Wu2021 replicate 1 ((d) without distance adjustment, (e) metacell result, (f) distance adjustment), GM12878-Wu2021 replicate 2 ((g) without distance adjustment, (h) metacell result, (i) distance adjustment), CD4+ T cells ((j) without distance adjustment, (k) metacell result, (l) distance adjustment) and CD14+ monocytes ((m) without distance adjustment, (n) metacell result, (o) distance adjustment) in PBMC-10X2021.

We then explored the actual signals in the multi-modal single cell data for both the positive and negative pairs from gold standard datasets. Sup. Figs. 12a, b display gene expression versus chromatin accessibility for gene *CDYL* and one of its enhancers (chr6:4,941,739-4,942,936). This pair is identified as a false positive pair according to the gold standard HiChIP-Bhattacharyya2019 and ChIA-PET-Li2019. A sizable correlation between the normalized expression and the accessibility of the pair is revealed among metacells obtained with SEACells and single cells in Sup. Figs. 12a, b. Next, we looked at a true positive pair, gene *MYL6* and enhancer chr12:56,129,186-56,129,691, according to the gold standard datasets EnhancerAtlas 2.0, HiChIP-Bhattacharyya2019 and ChIA-PET-Li2019. Sup. Figs. 12c, d depict lack of correlation for this pair both at the metacell and single cell level.

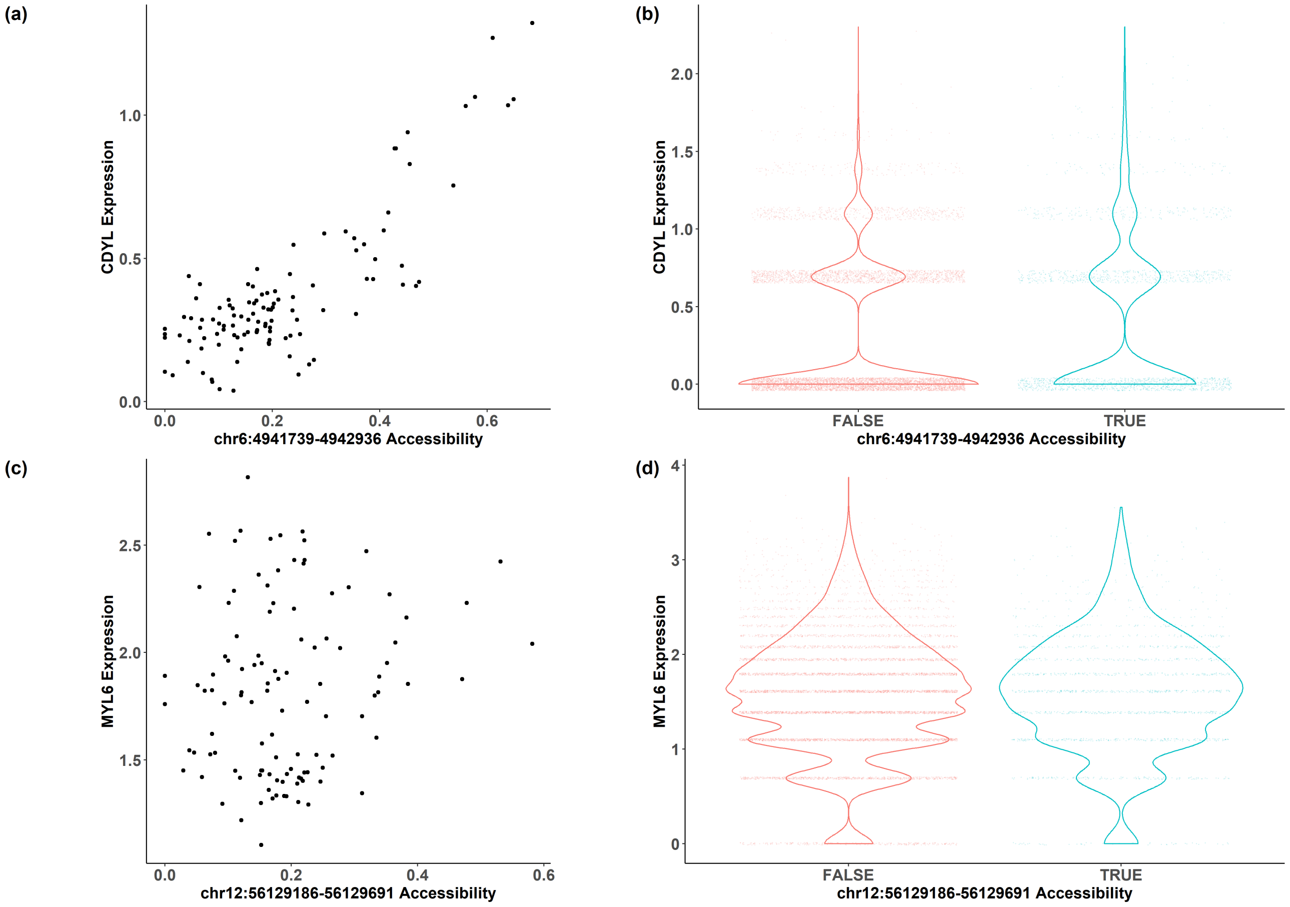

Figure 12: For a **false positive** pair (CDYL-chr6:4941739-4942936) according to the gold standards HiChIP-Bhattacharyya2019 and ChIA-PET-Li2019, CDYL expression vs. chr6:4941739-4942936 chromatin accessibility among K562-Xu2022 (a) metacells and (b) single cells (Wilcoxon rank sum test p-value $= 3.283 \times 10^{-12}$).
For a **true positive** pair (MYL6-chr12:56129186-56129691) according to the gold standards EnhancerAtlas 2.0, HiChIP-Bhattacharyya2019 and ChIA-PET-Li2019, MYL6 expression vs. chr12:56129186-56129691 chromatin accessibility among the K562-Xu2022 (c) metacells and (d) single cells (Wilcoxon rank sum test p-value = 0.9178).

12 Further investigation of clustering the cells by GEEES-inferred regulatory interactions

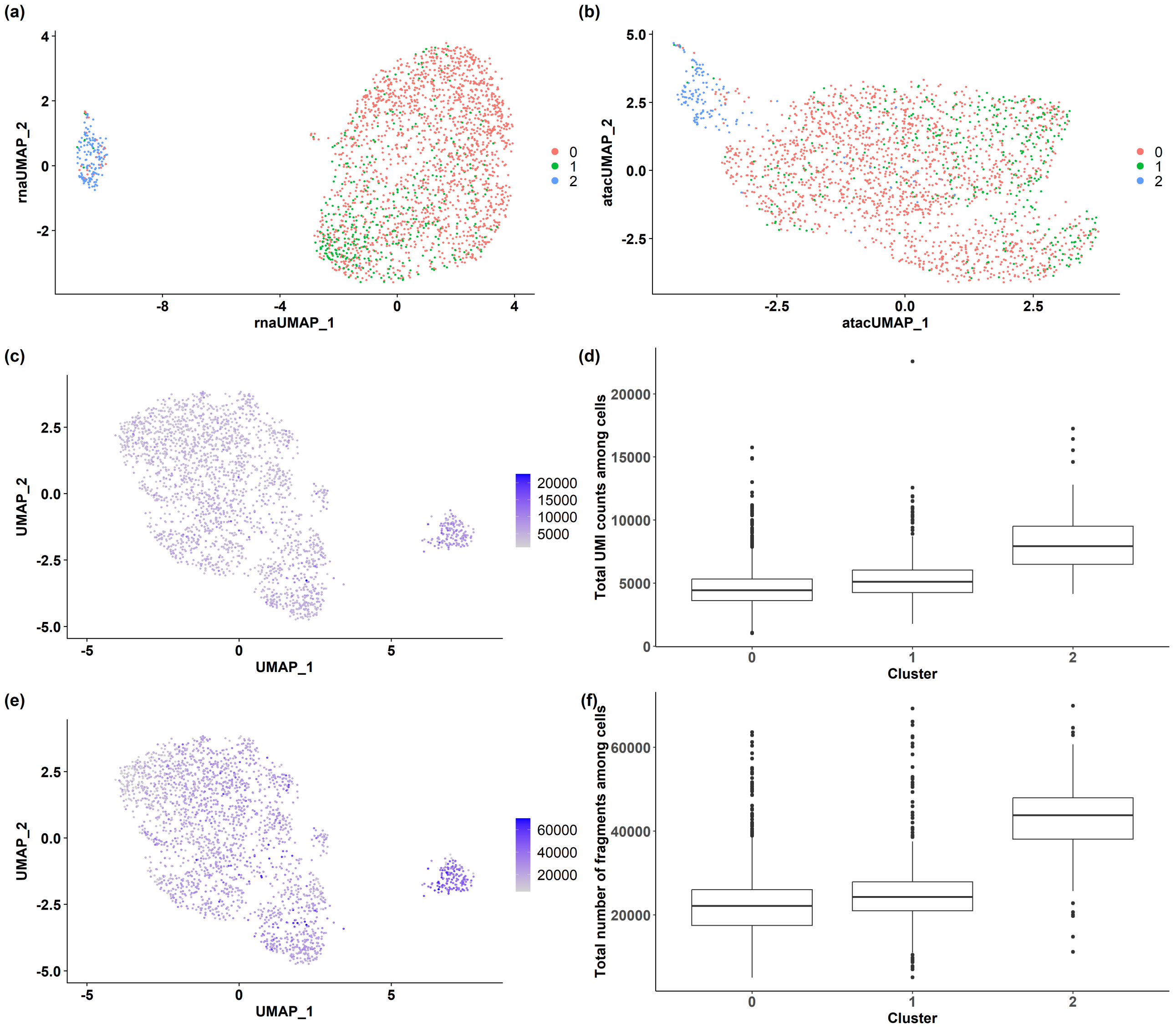

Figure 13: (a) UMAP visualization of CD14+ monocyte cells from PBMC-10X2021 based on their scRNA-seq expression normalized by `sctransform` (Hafemeister and Satija, 2019). Cells are colored by the clustering result of GEEES. (b) UMAP visualization of CD14+ monocyte cells from PBMC-10X2021 based on their scATAC-seq accessibility normalized by TFIDF. Cells are colored by the clustering result of GEEES. (c) UMAP visualization of CD14+ monocyte cells from PBMC-10X2021 using GEEES statistics for all gene-enhancer pairs as features, colored by total UMI counts in each cell. (d) Box plot of the total UMI counts for each cluster. (e) UMAP visualization of CD14+ monocyte cells from PBMC-10X2021 using GEEES statistics for all gene-enhancer pairs as features, colored by total number of fragments in scATAC-seq for each cell. (f) Box plot of total number of fragments in scATAC-seq for each cluster.

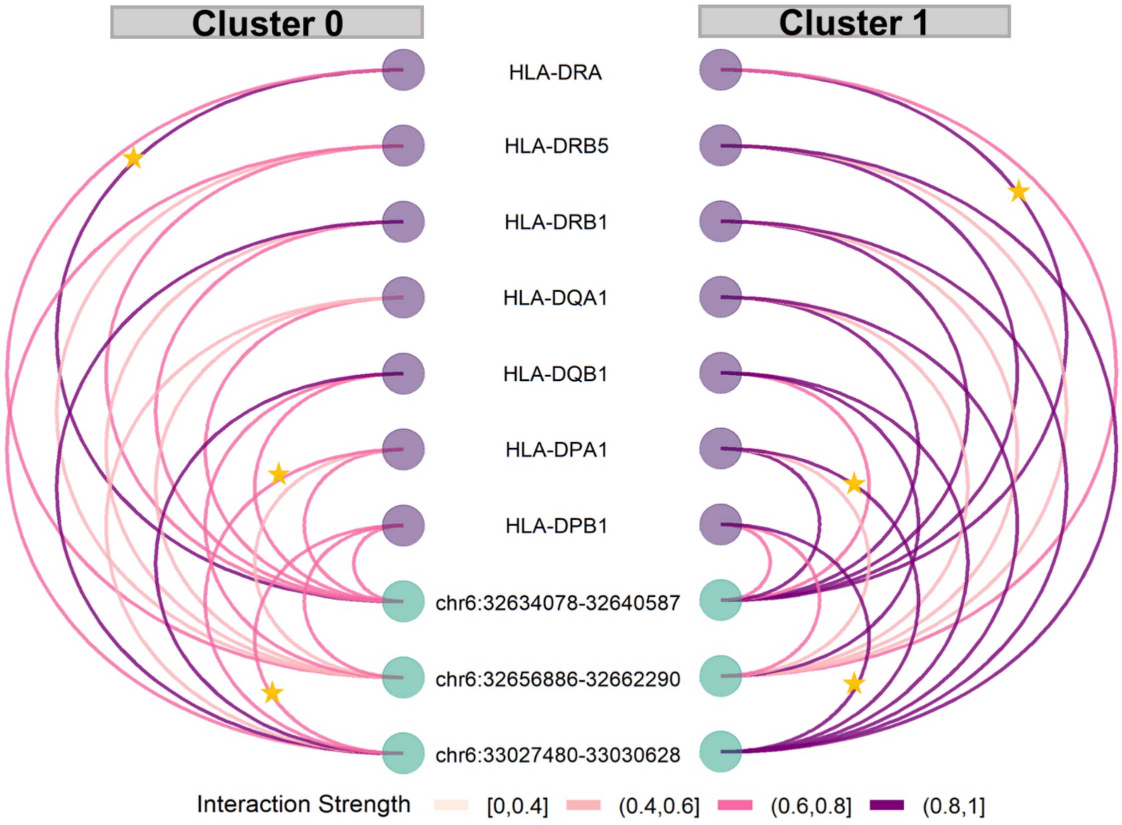

Figure 14: Normalized GEEES interaction strengths in cluster 0 and cluster 1 between HLA family genes and their enhancers. For gene g and enhancer e pair in cluster i, the normalized GEEES interaction strength is defined as $\text{GEEES}_{\text{Norm}}(gei) = \frac{\text{GEEES}(gei)}{max_{e' \in E_g, i' \in \{0,1\}} \text{GEEES}(ge'i')}$, where GEEES$(gei)$ is the median aggregation of g-e GEEES statistics among all cluster i cells. The gene-enhancer pairs validated by the pcHiC-Javierre2016 are marked by stars on the edges.

References

Andersson, R. *et al.* (2014). An atlas of active enhancers across human cell types and tissues. *Nature*, **507**(7493), 455–461.

Bhattacharyya, S. *et al.* (2019). Identification of significant chromatin contacts from hichip data by fithichip. *Nature communications*, **10**(1), 4221.

Chen, S. *et al.* (2019). High-throughput sequencing of the transcriptome and chromatin accessibility in the same cell. *Nature biotechnology*, **37**(12), 1452–1457.

De Rop, F. V. *et al.* (2023). Systematic benchmarking of single-cell atac-sequencing protocols. *Nature Biotechnology*, pages 1–11.

Duren, Z. *et al.* (2022). Regulatory analysis of single cell multiome gene expression and chromatin accessibility data with screg. *Genome biology*, **23**(1), 1–19.

Fang, R. *et al.* (2021). Comprehensive analysis of single cell atac-seq data with snapatac. *Nature communications*, **12**(1), 1337.

Fulco, C. P. *et al.* (2019). Activity-by-contact model of enhancer–promoter regulation from thousands of crispr perturbations. *Nature genetics*, **51**(12), 1664–1669.

Gao, T. and Qian, J. (2020). Enhanceratlas 2.0: an updated resource with enhancer annotation in 586 tissue/cell types across nine species. *Nucleic acids research*, **48**(D1), D58–D64.

Gasperini, M. *et al.* (2019). A genome-wide framework for mapping gene regulation via cellular genetic screens. *Cell*, **176**(1), 377–390.

Hafemeister, C. and Satija, R. (2019). Normalization and variance stabilization of single-cell rna-seq data using regularized negative binomial regression. *Genome biology*, **20**(1), 296.

Hecker, D. *et al.* (2023). The adapted activity-by-contact model for enhancer–gene assignment and its application to single-cell data. *Bioinformatics*, **39**(2), btad062.

Javierre, B. M. *et al.* (2016). Lineage-specific genome architecture links enhancers and non-coding disease variants to target gene promoters. *Cell*, **167**(5), 1369–1384.

Jiang, Y. *et al.* (2022). Nonparametric single-cell multiomic characterization of trio relationships between transcription factors, target genes, and cis-regulatory regions. *Cell Systems*, **13**(9), 737–751.

Kartha, V. K. *et al.* (2022). Functional inference of gene regulation using single-cell multi-omics. *Cell genomics*, **2**(9).

Li, G. *et al.* (2019). Chromatin interaction analysis with updated chia-pet tool (v3). *Genes*, **10**(7), 554.

Ma, S. *et al.* (2020). Chromatin potential identified by shared single-cell profiling of rna and chromatin. *Cell*, **183**(4), 1103–1116.

Mitra, S. *et al.* (2024). Single-cell multi-ome regression models identify functional and disease-associated enhancers and enable chromatin potential analysis. *Nature Genetics*, **56**(4), 627–636.

Moore, J. E. *et al.* (2020). A curated benchmark of enhancer-gene interactions for evaluating enhancer-target gene prediction methods. *Genome biology*, **21**(1), 1–16.

Pliner, H. A. *et al.* (2018). Cicero predicts cis-regulatory dna interactions from single-cell chromatin accessibility data. *Molecular cell*, **71**(5), 858–871.

Schraivogel, D. *et al.* (2020). Targeted perturb-seq enables genome-scale genetic screens in single cells. *Nature methods*, **17**(6), 629–635.
